# Bionic Nanostructures Create Mechanical Signals to Mediate the Composite Structural Bone Regeneration Through Multi‐System Regulation

**DOI:** 10.1002/advs.202502299

**Published:** 2025-06-04

**Authors:** Yangfan Pei, Yihan Wang, Jingxia Chen, Jing Zhou, Yuzhu Han, Xiuyu Liu, Siyu Chen, Sheng Chen, Dixin He, Yunxiao Wu, Huixin Lv, Yanmin Zhou

**Affiliations:** ^1^ Department of Oral Implantology Hospital of Stomatology Jilin University Changchun 130021 China; ^2^ Jilin Provincial Key Laboratory of Tooth Development and Bone Remodeling Hospital of Stomatology Jilin University Changchun 130021 China

**Keywords:** biomimetic, bone tissue engineering, mechanical signals, nanomaterials, scaffold

## Abstract

Regenerating bone defects has long been recognized as a significant clinical challenge. Drawing inspiration from the structure and properties of natural bone, bionic nanomaterials have emerged as a focal point in the field of bone tissue engineering. Unlike traditional scaffold materials, these advanced nanomaterials offer a remarkable capacity to replicate the intricate microenvironment of the stem cell niche. This ability facilitates enhanced migration, proliferation, and differentiation of stem cells, thereby promoting efficient new bone formation. Of particular significance is the application of contemporary nanotechnology, which enables the design of bone tissue engineering scaffolds with precisely tailored nanoscale characteristics. These include properties such as stiffness, pore size and porosity, nanomorphology, curvature, shear stress, viscoelasticity, hydrostatic pressure, and biochemical functionalities. Such customization affords precise control over stem cell behavior, guiding their cultivation or differentiation into desired phenotypes with spatial and temporal precision. Consequently, this approach significantly amplifies the efficacy of bone tissue regeneration. This article provides a comprehensive overview of the design principles and critical requirements for developing bionic nanomaterials as artificial stem cell niches. Furthermore, it consolidates current advancements in the field, examining various types of bionic nanomaterials and biomimetic technologies, alongside their diverse applications in bone tissue engineering.

## Graphical abstract

Inspired by the structural and functional characteristics of bone, bionic nanomaterials combined with nanotechnology can more accurately replicate stem cell niches, enabling the design of bone tissue engineering scaffolds with diverse nanoscale properties to promote stem cell migration, proliferation, and differentiation. This precise control over stem cell behavior substantially enhances bone regeneration.

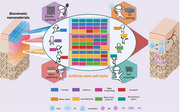

## 1 Introduction

Bone defects caused by severe trauma, tumor resection, or congenital deformities represent significant clinical challenges.^[^ ^1^, ^2^ ^]^ While autologous and allogeneic bone grafting remain the gold standards for treating such defects,^[^ ^3^ ^]^ these approaches face numerous limitations, including donor site pain, necrosis, neurovascular injury, donor shortages, immune rejection, and the risk of infection.^[^ ^4^, ^5^ ^]^ To address these issues, bone tissue engineering (BTE) has emerged as a promising alternative to guide bone regeneration.^[^ ^6^ ^]^ Traditionally, BTE has focused on the development of implants utilizing scaffolds, cells, mechanical stimuli, and soluble factors.^[^ ^7^ ^]^ However, while traditional BTE can successfully induce new bone formation, the regenerated bone often differs substantially from natural bone in structure and mechanical properties, leading to long‐term remodeling and potential resorption.

In recent years, biomimetic approaches in BTE have garnered significant attention.^[^ ^8^ ^]^ These methods aim to replicate the complex microenvironment of natural bone tissue by employing biomaterials designed to mimic the composition, structure, and function of the three‐dimensional (3D) extracellular matrix (ECM).^[^ ^9^ ^]^ More than 30 years ago, Schofield introduced the concept of the “niche,” describing the specialized microenvironment that regulates the homeostasis of hematopoietic stem cells (HSCs).^[^ ^10^ ^]^ This niche governs stem cell behavior through direct intercellular contact, as well as the production and presentation of growth factors, cytokines, chemokines, and ECM proteins that interact with stem cells.^[^ ^11^ ^]^ Similarly, biomimetic scaffolds aim to guide stem cells to grow and differentiate into appropriate phenotypes at the right time and location by closely replicating the natural bone microenvironment.^[^ ^12^ ^]^ Furthermore, these scaffolds incorporate physical, mechanical, and biochemical properties that significantly influence the cellular environment, offering fine‐tuned control over cellular processes. Biomimetic strategies hold tremendous potential to enhance bone regeneration while addressing the limitations of traditional approaches.

While the macro‐ and micro‐architectures of bone have been effectively replicated through porous scaffolds, achieving a complete simulation of bone tissue requires nanoscale control over material distribution. The seamless integration of micro‐ and nanoscale elements is critical to developing biomimetic structural biomaterials.^[^ ^13^ ^]^ In this context, nanomaterials have emerged as a transformative platform for bone tissue regeneration. Possessing a high surface‐area‐to‐volume ratio, nanomaterials can be precisely engineered to replicate the nanoscale structure and biochemical cues of natural ECM.^[^ ^14^, ^15^ ^]^ Concurrent advances in bionanotechnology have further revolutionized orthopedic treatments by more accurately mimicking the hierarchical structure, composition, biomineralization, mechanical properties, cellular interactions, biochemical activities, and endogenous signaling pathways of natural tissues.^[^ ^16^ ^]^ These innovations bring us closer to creating complex, multidimensional models of stem cell niches, recreating natural microenvironments in vitro, and manipulating niches in vivo to regulate stem cell functions.^[^ ^17^ ^]^

This paper provides an overview of the latest developments in artificial stem cell niches created using biomimetic nanomaterials in BTE. First, it introduces the fundamental principles of bone physiology. Next, it outlines the design strategies and basic requirements for biomimetic nanomaterials in vitro. Additionally, the paper reviews various types of biomimetic nanomaterials and nano‐biomimetic technologies and discusses their applications in simulating natural bone structure for BTE. Finally, it identifies the challenges associated with biomimetic nanomaterials for artificial stem cell niche construction, while exploring future prospects and research directions.

## 2 Fundamentals of Bone Physiology

A more detailed and realistic simulation of bone stratification is critical for achieving successful bone tissue regeneration. Various manufacturing techniques enable precise control over the shape and mechanical properties of biomimetic nanomaterials, ensuring they meet the inherent mechanical and biochemical requirements of diverse bone defects and tissues. This tailored approach ensures optimal support and seamless integration, thereby maintaining the long‐term stability and functionality of regenerated bone. Mature lamellar bone exhibits a layered, anisotropic structure that can be analyzed across multiple hierarchical levels. In 1988, Weiner and Wagner pioneered the classification of natural bone tissue into seven distinct hierarchical levels. Since then, various classification schemes have emerged, ranging from four to nine levels.^[^ ^18^, ^19^ ^]^ More recently, in 2018, this classification was expanded to encompass twelve structural levels specific to lamellar bone types.^[^ ^20^ ^]^ These frameworks aim to elucidate bone structure comprehensively, spanning scales from the entire bone to the microscopic and nanoscale levels.^[^ ^19^ ^]^ This section provides a detailed examination of bone structure, offering crucial insights into the design of biomimetic nano‐scaffolds.

**Figure 1:**
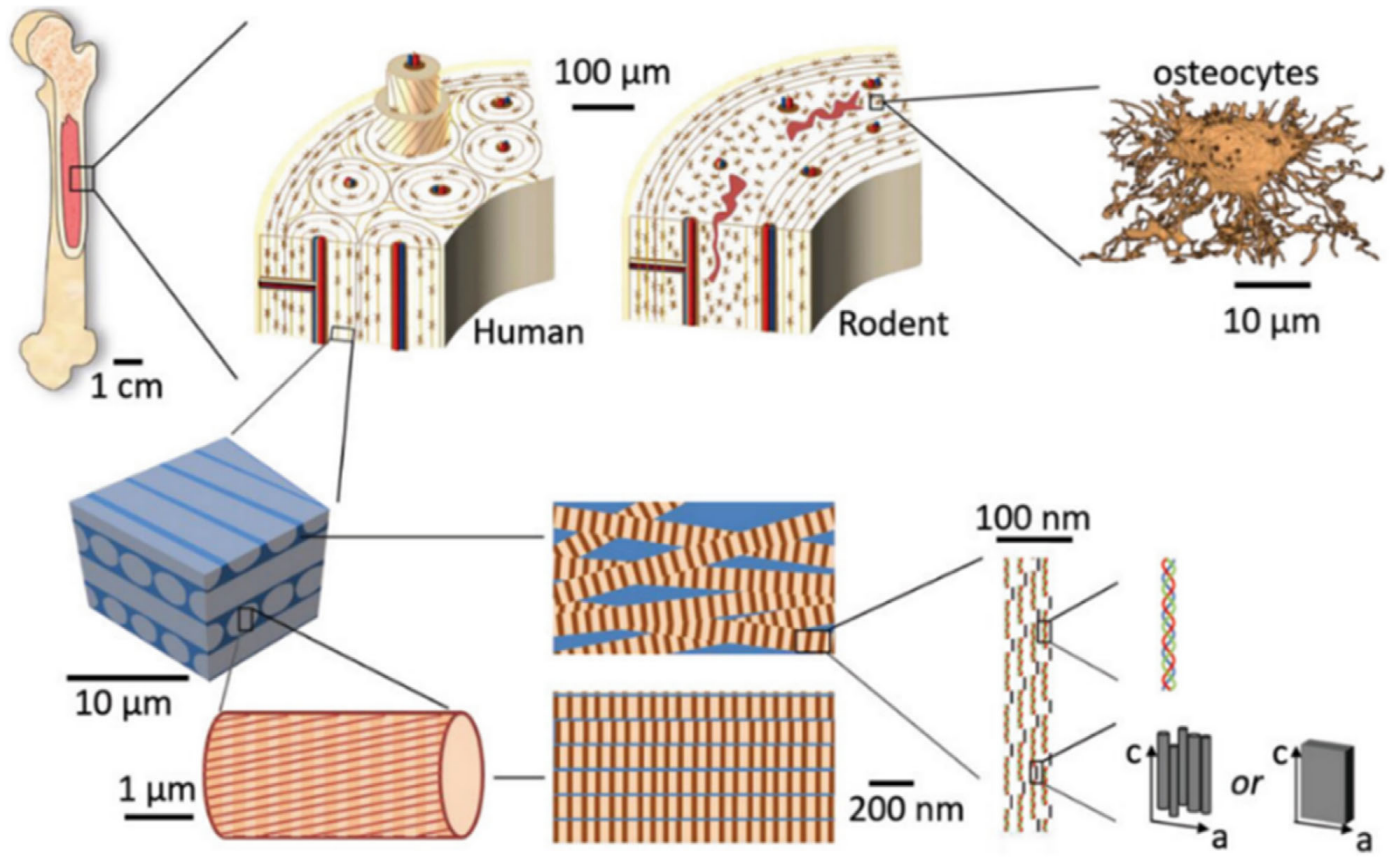
Bone structure. Reproduced with permission.^[^ ^21^ ^]^ Copyright 2023, Wiley‐VCH.

Bone is hierarchically organized, encompassing structures that span the macroscopic scale (measured in centimeters and millimeters) to the nanoscale (**Figure** **1**).^[^ ^21^ ^]^ At the macroscopic level, bone tissue comprises the periosteum, bone marrow, and bone itself.^[^ ^22^ ^]^ The periosteum is a specialized, highly vascularized connective tissue that covers nearly all bone surfaces except for joints. It consists of an outer fibrous layer and an inner cambium layer. This dynamic environment provides a niche for multipotent stem cells and a reservoir of molecular factors that regulate cellular behavior.^[^ ^23^, ^24^ ^]^ Within the bone's core, the bone marrow harbors hematopoietic stem cells capable of differentiating into red blood cells, white blood cells, or platelets.^[^ ^25^ ^]^ At the nanoscale, bone comprises numerous structural proteins and polysaccharides, with collagen fibers serving as the primary component. These fibers, with diameters ranging from 35 to 60 nm and lengths up to 1 µm, exhibit a periodicity of 67 nm and inter‐fibril spacing of ≈40 nm.^[^ ^26^, ^27^, ^28^ ^]^ Collagen fibers are formed through the self‐assembly of unique triple‐helix biomolecules.^[^ ^13^, ^29^ ^]^ These fibrils are mineralized with hydroxyapatite crystals, an anisotropic and mechanically robust inorganic component, which occupies the gaps within the collagen framework.^[^ ^30^, ^31^ ^]^ The triple helical structure of the protocollagen molecule enables mineralized collagen fibers (MCF) to undergo elastic deformation through sliding mechanisms. The deposition of hydroxyapatite (HA) within and on collagen fibers significantly enhances bone strength and toughness. Factors such as the density or volume fraction, cross‐arrangement, orientation, and morphology of HA crystals, along with the orientation of MCF and enzymatic cross‐linking in bone, play a crucial role in optimizing the mechanical properties of bone.^[^ ^32^, ^33^ ^]^

Bone can be divided into two types: i) Woven (primary) bone, a transient structure formed during early development or repair, is characterized by an irregular matrix of interwoven trabeculae. Its porosity ranges from 50% to 90%, with a compressive strength of 2–12 MPa.^[^ ^34^ ^]^ The trabeculae align along stress lines, enhancing mechanical strength. Spaces within trabecular bone often house red marrow, essential for haematopoiesis.^[^ ^35^, ^36^ ^]^ ii) Lamellar (secondary) bone, a denser, more organized structure with lower porosity (5%–10%) and higher compressive strength (100–180 MPa), replaces woven bone. Thin lamellae, ≈3 µm thick, are arranged concentrically around vertical Haversian canals, which house blood vessels and nerves. This system, known as the osteon or Haversian system, forms through osteoclastic resorption followed by osteoblastic deposition.^[^ ^18^ ^]^ The alternating orientation of collagen fibers in adjacent lamellae provides resistance to torsional forces. Osteocytes, situated within lacunae between lamellae, are interconnected by canaliculi, forming an intricate communication network.^[^ ^27^ ^]^

Bone tissue layer simulation relies on precise nano‐scale control of material distribution. Indeed, the accurate combination of micro‐ and nanoscale aspects of bone structure forms the foundation for developing truly biomimetic structural materials. Nanoscale features can serve as effective enhancers for producing nanomaterials with superior mechanical properties and stability. The biomimetic porous layered structure of nanomaterials, engineered at macro/micro/nano scales (with particular emphasis on the nano scale), creates a favorable microenvironment that regulates cellular activities—such as adhesion, migration, proliferation, and differentiation—and promotes bone ingrowth, integration, and vascularization.

## 3 Cell Microenvironment Design Strategies and Fundamental Requirements

Stem cell niches constitute a specialized microenvironment that governs the quiescence, self‐renewal, and functional behavior of mesenchymal stem cells (MSCs). These niches play a pivotal role in regulating stem cell migration and differentiation in response to physiological needs. Niches regulate stem cell behavior through direct cell‐to‐cell contact between stem cells and adjacent niches, as well as through the production and presentation of growth factors and extracellular matrix (ECM) proteins that interact with stem cells. The construction of ideal biomimetic nano scaffolds requires precise physical and mechanical properties, enabling stem cells to recognize and respond appropriately to these cues. Additionally, biochemical signals are an essential component of the ecological niche, facilitating communication and information exchange between cells. The physicochemical characteristics of the cellular nanoenvironment significantly influence stem cell behavior, and understanding these interactions holds profound implications for controlling and programming various cellular functions. In vitro models that incorporate these features will enable detailed studies of stem cell behavior, ultimately allowing for precise control over artificial scaffolds to direct stem cell growth and differentiation. Regulatory signals within the niche can be broadly categorized into mechanical signals and biochemical signals, each of which contributes to the dynamic interplay governing stem cell fate. This section explores the design strategies and fundamental requirements associated with these two signal types.

### 3.1 Mechanical Signals

Biomimetic nanomaterials facilitate the migration, proliferation, and differentiation of bone marrow‐derived mesenchymal stem cells (BMSCs) through mechanical cues. These cues include stiffness, pore size and porosity, nanoscale morphology, curvature, fluid shear stress, viscoelasticity, and hydrostatic pressure. Mechanical signals are transmitted to cells via mechanotransduction pathways, which convert extracellular mechanical stimuli into intracellular biochemical responses, thereby influencing cellular function (**Table** **1**).

**Table 1:** Nanoscale mechanical properties regulate stem cell behavior.

Mechanical signal	Material	Mechanism	MSC type	Refs.
Stiffness	RGD modified agarose, polyethylene glycol dimethacrylate	Integrin‐adherence‐ligand bond	Murine MSC	[37]
	CAVM hydrogel	YAP/TAZ	Rats BMSC	[38]
	Nanoparticle mineralized collagen glycosaminoglycan (MC‐GAG) scaffold	Wnt (cWnt)	Primary human bone marrow‐derived MSC	[39]
Pore size, porosity	Poly (L‐lactic acid) (PLLA) (<125µm) nanofiber	Maintaining the stem cell phenotype is associated with increased expression of Gli1	Primary suture MSC	[40]
	RGD Modified alginate Gel (120µm)	N‐cadherin mediates intercellular interactions, enhancing the paracrine signaling of mesenchymal stem cells (MSCs)	Rat BMSC	[41]
	Pickering emulsion templates containing AGNPs and Cu‐HA	NF‐κB and MAPK	BMSCs from SD rats	[42]
Nanoscale morphology	PLN	Activate YAP and antagonize NF‐κB	Mice BMSC	[43]
	Electrospinning poly‐L‐Lactide (PLLA)	miR‐193a‐3p‐MAP3k3 Signal axis	Human BMSC	[44]
	Stereotopological Silicon nanowires (SiNWs)	The α2/β1 integrin heterodimer and the focal adhesion molecules pFAK and vinculin	Human MSC	[45]
	Poly (lactic acid‐glycolic acid copolymer)/fish collagen/Nano‐hydroxyapatite (PFCH)	HIF‐1 α	BMSC	[46]
	HAP integrated polyvinylidene fluoride (P (VDF‐TrFE))	Maculin and paracrine	Osteoblast	[47]
	Polycaprolactone	Signaling of Wnt, Notch and HH	hASC	[48]
Curvature	PLGA microspheres	Enhanced F‐actin and Lamin A	rat BMSC	[49]
	PDMS substrate	SMAD path	MC3T3‐E1	[50]
	β‐TCP nanoparticles and 1,6 hexadiol diacrylate based resin∖	Integrin‐mediated FAK and mitogen‐activated protein kinase (MAPK) pathways∖	human MSC	[51]
	Enantiomer lipid mimics glutamate Derivatives (L/D‐UG)	Promote Itgβ1 aggregation	Encapsula‐ ted MSC	[52]
Fluid shear stress	External flow chamber	β‐catenin/Wnt	Human BMSC	[53]
	New shear stress loading device (Thailand Patent No. 1 801 000 629)	Cx43 and downstream Erk1/2	Mouse iPSC	[54]
	SF internal perfusion bioreactor	–	Human MSC	[55]
	Laminar flow perfusion bioreactor	Rho‐ROCK	Rat BMSC	[56]
	Polylysine coated slide parallel plate flow system	BMP‐2	MC3T3‐E1 and mouse BMSC	[57]
Viscoelasticity	Rgd‐coupled alginate saline gel	Integrin adhesion, local aggregation of RGD ligands, actomyosin contractility, and nuclear localization of YAP	Mouse BMSC	[58]
	Rgd‐coupled alginate saline gel	ROCK and Rac1 channels	D1 MSC, hBM‐MSC, hDPSC	[59]
	Borate based hydrogel	YAP/TAZ	hMSC	[60]
	Chitin whisker assembled polymers (L‐lactide)	Integrin and HIF‐1α	BMSC	[61]
Hydrostatic pressure	Sodium alginate	PI3K/Akt	Rat BMSC	[62]
	Airtight acrylic cell culture chamber	ERK1/2 and p38 MAPK	UE7T‐13	[63]
	Hydrostatic pressure instrument	RhoA and Rac1	Rat BMSC	[64]
	Resistant backpressure regulator	PAGBC/miR‐133b/RUNX2	AMSC	[65]

The ability to engineer mechanical properties at the nanoscale offers unprecedented control over the cellular microenvironment. For example, matrix stiffness directly affects lineage commitment, with softer substrates promoting adipogenic differentiation and stiffer substrates favoring osteogenesis. Similarly, pore size and porosity are critical for regulating nutrient diffusion and cell infiltration, while nanoscale morphology and curvature impact cell adhesion and migration. Additionally, dynamic forces such as fluid shear stress and hydrostatic pressure mimic physiological conditions, further enhancing the biomimetic potential of engineered niches.

#### 3.1.1 Stiffness

The intrinsic stiffness of the extracellular matrix (ECM) plays a pivotal role in regulating cell morphology, proliferation, and fate, ultimately influencing the osteogenic differentiation of mesenchymal stem cells (MSCs) and promoting bone repair.^[^ ^66^ ^]^ Notably, the response of MSCs to stiffness and timing differs significantly between 3D and 2D environments, as evidenced by the degree of nuclear envelope wrinkling.^[^ ^67^ ^]^ Stem cells discern the dimensionality of their extracellular environment through the constrained shaping of their cytoskeleton.^[^ ^68^ ^]^ In a seminal study, Engler et al. demonstrated that human MSCs cultured on polyacrylamide hydrogels with stiffness simulating brain tissue rigidity (0.1–1.0 kPa) exhibited neurogenic differentiation. When the stiffness was increased to approximate bone tissue rigidity (25–40 kPa), the cells underwent osteogenic differentiation and adopted morphologies resembling those of mature osteoblasts.^[^ ^69^ ^]^ On rigid matrices, stem cells formed pronounced polygonal shapes with distinct adhesive protein bundles, contrasting with the rounded morphologies observed on softer matrices.^[^ ^70^ ^]^

In 3D environments, however, static high‐stiffness hydrogels tend to limit integrin rearrangement, cell diffusion, and volumetric expansion, thereby constraining MSC osteogenic differentiation.^[^ ^71^ ^]^ Conversely, dynamic stiffening of 3D matrices facilitates increased cell spreading and volumetric expansion. Following secondary cross‐linking, high stiffness enhances material mechanical strength, and preconditioning cells in soft matrices prior to stiffening promotes osteogenic differentiation.^[^ ^72^, ^73^ ^]^ MSCs thus exhibit a preference for biomimetic, dynamic microenvironments, and their response to stiffness is highly time‐dependent. The high stiffness of the nanocomposite network can be attributed to the chemical functionality of the nanoparticles, which facilitates the cross‐linking of multiple polymer chains to the nanoparticle surface. This cross‐linking allows for precise customization of the hydrogel network properties while maintaining its pore morphology and internal structure.^[^ ^74^ ^]^ Furthermore, under the mechanical property gradient in nanocomposites, scaffolds have the potential to influence cell morphology and promote differentiation along distinct regions.^[^ ^75^ ^]^

**Figure 2:**
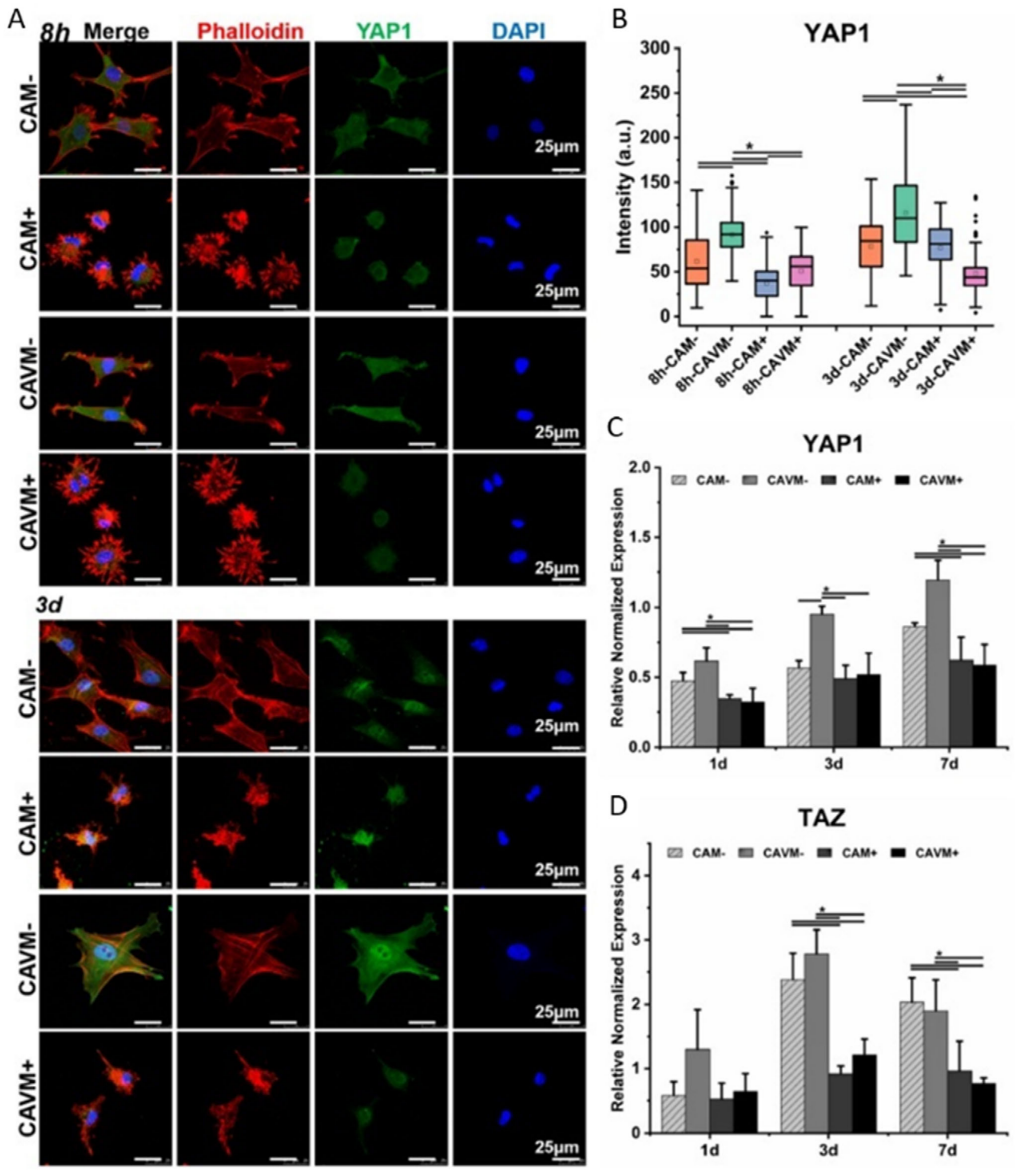
Effect of actin depolymerization on YAP1 and TAZ expression of BMSCs on CAM and CAVM with Cyto D (CAM+, CAVM+) or without Cyto D (CAM‐, CAVM‐) treatment. A) Phalloidin (red)/DAPI (blue) staining as well as immunofluorescence staining of YAP1 (green), B)corresponding immunofluorescence intensity analysis of YAP1, and quantitative analysis of C) YAP1 and D) TAZ gene expressed by BMSCs (*n* = 3, * *p* < 0.05). Reproduced with permission.^[^ ^80^ ^]^ Copyright 2021, Elsevier.

Mechanistically, stiffness regulates osteogenesis through the activation of mechanotransduction mediators such as YAP/TAZ and the canonical Wnt (cWnt) signaling pathway.^[^ ^39^, ^67^, ^76^ ^]^ Stiff matrices promote osteogenic differentiation via integrins, which form large protein complexes known as focal adhesions (FAs) and activate various pathways, including FAK, ROCK, Rho, and PIP5K‐PIP2, alongside transcriptional regulators such as YAP/TAZ and Runx2.^[^ ^77^, ^78^, ^79^ ^]^ For example, collagen hydrogels modified with alkaline phosphatase and phosphorylated molecules (CAVM hydrogels) enhanced MSC osteogenesis. The cells exhibit robust diffusion and maintain a cytoskeleton with increased tension, which facilitates the nuclear localization and activation of YAP/TAZ. Notably, treatment with the actin polymerization inhibitor Cyto D inhibits cell migration and disrupts stress fibers, thereby impairing the nuclear localization and retention of YAP/TAZ. (**Figure** **2**)^[^ ^80^ ^]^ Additionally, YAP mediates feedback mechanisms that maintain favorable metabolic states, with mitochondria exhibiting elongation and fusion on rigid ECM. However, ECM stiffness exceeding 80 kPa diminishes mitochondrial regulation.^[^ ^81^ ^]^ Interestingly, while classical Wnt/β‐catenin signalling may inhibit osteogenesis on stiffer matrices, non‐canonical Wnt5a has been shown to promote this process.^[^ ^39^, ^82^ ^]^

#### 3.1.2 Pore Size and Porosity

Pore size refers to the dimensions of the voids within a material, while porosity describes the proportion of void space within a given volume of the scaffold. Modern manufacturing techniques allow precise control of pore size in porous scaffolds.^[^ ^83^, ^84^ ^]^ Porous surfaces enhance mechanical interlocking between the scaffold and surrounding tissues, thereby improving the mechanical stability of implants.^[^ ^85^ ^]^ Both porosity and pore size significantly influence stem cell behavior.^[^ ^86^, ^87^ ^]^ An open, porous, and interconnected network is essential for promoting cell nutrition, proliferation, migration, tissue vascularization, and the formation of new tissue.^[^ ^88^, ^89^ ^]^ The importance of nanostructures in scaffolds cannot be overlooked. The integration of nanostructures within scaffolds not only provides a larger surface area for protein adsorption but also allows surface and interfacial nanostructures to modulate the conformation of growth factors, thereby enhancing their biological activity. This optimization can lead to improved osteogenic differentiation capabilities, which are critical for effective bone regeneration.

Cells cultured in vitro on scaffolds respond to pore size in determining their differentiation fate. Thus, variations in cell and tissue outcomes are often attributed to the geometric configuration of scaffold pores.^[^ ^90^ ^]^ Large pores create a favorable niche for bone formation, whereas small pores (diameter < 125 µm) maintain cells in a quiescent state, preventing differentiation.^[^ ^40^ ^]^ Pores ranging from 10 to 200 µm can facilitate N‐cadherin‐mediated cell‐cell and cell‐matrix interactions, enhancing the paracrine effects of mesenchymal stem cells (MSCs).^[^ ^41^ ^]^ Additionally, nanoscale pores improve cell adhesion, further promoting cellular functions.^[^ ^91^ ^]^ Chu et al. successfully developed a multifunctional, layered, interconnected porous scaffold using amine gelatin nanoparticles (AGNPs) and copper hydroxyapatite (Cu‐HA) nanoparticles as stabilizers. This scaffold featured porous nanotopological interfaces that increased the number of cell adhesion plaques and strengthened cell adhesion. Additionally, its high porosity facilitated cell recruitment, proliferation, and immune response modulation.^[^ ^42^ ^]^ Scaffolds with fixed pore sizes but higher porosity have been shown to boost MSC proliferation, migration, and osteogenic differentiation.^[^ ^92^, ^93^ ^]^ For instance, high‐temperature‐demanding protein A3 (HtrA3) was found to degrade collagen IV, increasing pore volume and enhancing integrin β1 expression, providing anchorage for MSC migration.^[^ ^94^ ^]^ Moreover, higher porosity increases the specific surface area, offering more sites for protein adsorption and enabling enhanced interaction with osteogenic‐related proteins, which promotes cellular osteogenic functionality and new bone tissue formation. A larger specific surface area also accelerates the release of degradation products, further supporting osteogenesis.^[^ ^95^ ^]^ However, the precise mechanisms through which pore size and porosity regulate MSC behavior remain to be elucidated.

#### 3.1.3 Nanomorphology

The interaction between the nanoscale structures of cells and the extracellular matrix (ECM) is highly complex, with nanoscale topography at the interface significantly influencing cell behavior.^[^ ^96^, ^97^ ^]^ Previous studies have demonstrated how cells respond to environmental cues, showing, for instance, that certain nanoscale‐structured surfaces can elicit ultrahigh adhesion. This is attributed to the periodic arrangement of layered nanostructures, which enhances the scaffold's surface area and wettability, creating optimal conditions for cell adhesion.^[^ ^98^, ^99^ ^]^ Moreover, cells utilize nanoscale processes, such as lamellipodia and filopodia, for movement and adhesion to substrates. Research indicates that filopodia, with diameters ranging from 250 to 400 nm, play a key role in sensing and environmental exploration.^[^ ^100^, ^101^ ^]^ Consequently, the nanoscale morphology of scaffolds is crucial for regulating cell growth, adhesion, proliferation, and differentiation, underscoring its importance in tissue engineering and regenerative medicine.^[^ ^102^, ^103^, ^104^ ^]^

Dalby et al. conducted groundbreaking research demonstrating the critical influence of nanoscale topography on MSC population dynamics and bone‐specific differentiation.^[^ ^97^ ^]^ Since then, extensive studies have underscored the pivotal role of nanomorphology in the stem cell niche. For example, Shen et al. employed surface‐oriented epitaxial crystallization to create adaptive nanoscale topographies, represented as polycaprolactone (PCL) sheets, on 3D‐printed PCL scaffolds. This innovative approach provided precise control over hydroxyapatite nucleation, resulting in enhanced biomimetic mineralization. The adaptive nanoscale terrain established a biologically active microenvironment resembling the extracellular matrix (ECM), effectively guiding cell behavior and promoting new bone growth. In vitro analyses using MC3T3 cells confirmed the scaffold's superior biocompatibility, proliferation capacity, and osteogenic potential, further validating the efficacy of nanoscale topography in biomimetic mineralization.^[^ ^105^ ^]^ Additionally, researchers have successfully fabricated hierarchical, intrafibrillarly mineralized collagen (HIMC) with intricately interwoven nanoscale structures and surface chemistry closely resembling natural mineralized collagen.^[^ ^102^, ^103^, ^106^ ^]^ Cells cultured on HIMC displayed highly branched “osteoblast‐like” morphologies, with elongated filopodia and robust stress fiber formations. These findings suggest that the “ingenious” design of materials for bone tissue engineering should prioritize mimicking the natural nanoscale morphology of bone, specifically the layered nanostructures formed by the co‐assembly of collagen (Col) and nano‐hydroxyapatite (nano‐HA).

Nanomorphology plays a pivotal role in modulating the immune microenvironment to enhance osteogenesis. He et al. engineered highly‐oriented periodic lamellae poly(epsilon‐caprolactone) electrospun nanofibers (PLN) that provide topographical cues capable of regulating macrophage polarization. This regulation occurs via the activation of YAP, which interacts with IKK‐α and subsequently inhibits the NF‐κB signaling pathway. Western blot results demonstrated significant downregulation of IKK‐α expression and upregulation of IκB‐α expression. IκB‐α inhibits NF‐κB activation by sequestering it in the cytoplasm, while IKK‐α promotes the degradation of IκB‐α. The migration and osteogenic differentiation of bone marrow stromal cells were significantly enhanced by the direct interaction between IKK‐α and its ability to antagonize the NF‐κB signaling pathway.^[^ ^43^ ^]^ Additionally, nanomorphology facilitates osteogenesis through the activation of multiple signaling pathways, including the miR‐193a‐3p‐MAP3K3 axis,^[^ ^44^ ^]^ integrin‐mediated focal adhesion molecule formation,^[^ ^45^ ^]^ HIF‐1α,^[^ ^46^ ^]^ and key developmental pathways such as Wnt, Notch, and Hedgehog (HH) signaling.^[^ ^48^ ^]^

#### 3.1.4 Curvature

Surface curvature quantitatively describes the geometric characteristics of the cellular environment and is defined by two key metrics: mean curvature (H) and Gaussian curvature (K).^[^ ^107^ ^]^ Studies suggest that cells respond to surface curvatures comparable to or greater than their size.^[^ ^108^ ^]^ Werner et al. reviewed that cells can sense curvature at subcellular length scales (primarily through focal adhesion placement and growth) as well as at scales equal to or exceeding their own size, where stress fiber interactions with the cell nucleus play a pivotal role.^[^ ^109^ ^]^ For instance, mesenchymal stem cells (MSCs) cultured on hemispherical surfaces exhibit distinct behaviors: convex curvatures promote osteogenic gene expression, while concave curvatures enhance migration speed.^[^ ^110^ ^]^ This differential response highlights the significant role of curvature in influencing cell differentiation.^[^ ^111^, ^112^, ^113^ ^]^

Earlier research on MSCs in 2D cultures showed that cells on convex edges preferentially differentiate into the osteogenic lineage.^[^ ^114^ ^]^ More recent studies have shifted focus to complex 3D environments. Callens et al. examined preosteoblasts on polydimethylsiloxane (PDMS) scaffolds with varying curvatures. They found that cells preferred surfaces with at least one negative principal curvature (e.g., concave saddle‐like surfaces). The study also emphasized the importance of multicellular cooperation, where cells collectively modify their local environments to overcome unfavorable curvatures.^[^ ^115^ ^]^ Werner et al. further introduced the concept of “curvature sensing,” suggesting that MSCs adapt their migration behavior to avoid certain curvatures.^[^ ^116^ ^]^ Changes in surface curvature may induce cytoskeletal remodeling, leading to contractile forces generated by myosin, which drive cell diffusion and migration.^[^ ^117^, ^118^ ^]^ Notably, scaffolds made from β‐tricalcium phosphate (β‐TCP) with triply periodic minimal surfaces (TPMS) significantly enhance the osteogenic differentiation and paracrine activity of human MSCs (hMSCs). This effect is mediated by nuclear deformation into a contractile bean‐like shape.^[^ ^51^ ^]^ In addition to the curvature shape, the curvature magnitude is also crucial. Swanson et al. demonstrated that smaller spherical pores with curvatures in the range of 16.0–33.3 mm⁻¹ maintained MSC stemness, whereas larger pores with curvatures between 4.7 and 8.0 mm⁻¹ promoted osteogenic differentiation. However, the combined effects of curvature magnitude and shape on MSC osteogenesis remain insufficiently studied.^[^ ^86^ ^]^

**Figure 3:**
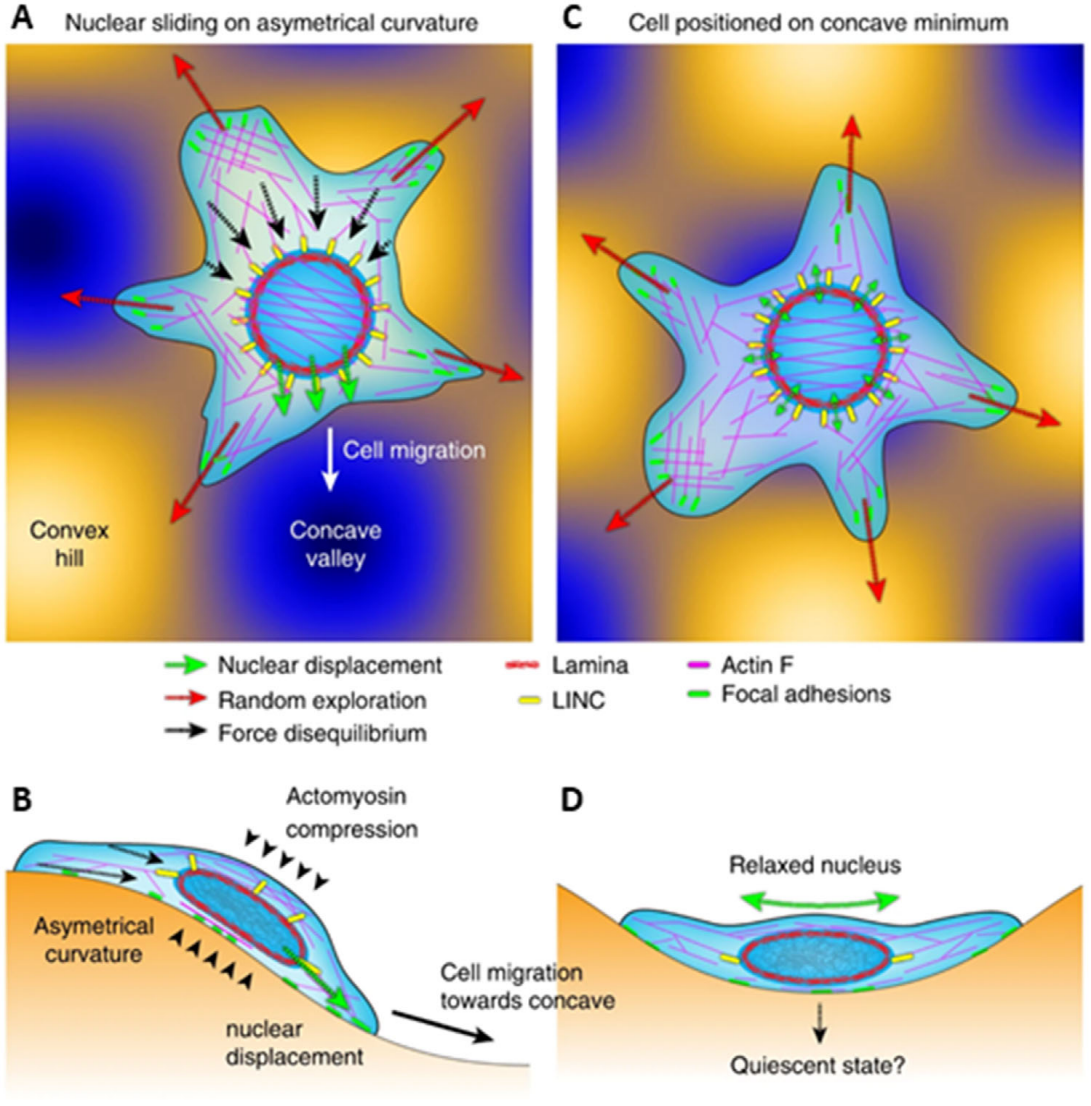
Curvature‐guided cell migration model. A,C) Asymmetric curvature: A) Adhesion triggers the nucleus to slide toward the concavity, altering the cell's random walk and resulting in cell localization in the concave valley C). B,D) Side views show the compression of the nucleus by actin and its relaxation on the concave curvature. Reproduced with permission.^[^ ^122^ ^]^ Copyright 2018, Springer Nature.

Curvature activates various mechanotransduction pathways.^[^ ^119^ ^]^ For example, surfaces with high principal curvature increase the phosphorylation of yes‐associated protein (YAP), while lower curvature suppresses YAP phosphorylation and promotes its nuclear translocation, initiating transcriptional activity toward osteogenic differentiation.^[^ ^86^ ^]^ Additionally, integrin‐mediated focal adhesion kinase (FAK) and mitogen‐activated protein kinase (MAPK) pathways^[^ ^51^ ^]^ are involved in curvature‐induced osteogenesis. Mechanosensitive ion channels like Piezo1^[^ ^120^ ^]^ are also activated by matrix‐induced membrane tension, contributing to osteogenic responses.^[^ ^121^ ^]^ Furthermore, the Arp2/3 complex and lamin A act as mechanosensors that influence cell migration toward concave curvatures (**Figure** **3**).^[^ ^122^, ^123^ ^]^ Despite these advances, further research is needed to understand how curvature impacts MSC proliferation and differentiation comprehensively, particularly in relation to complex signaling pathways and synergistic interactions.

#### 3.1.5 Fluid Shear Stress

Fluid shear stress (FSS), the frictional resistance generated by the flow of fluids such as blood or interstitial fluid, plays a pivotal role in regulating cellular gene expression and functional phenotypes. Interstitial fluid (ISF) flows through small channels connecting osteocytes to the mineralized bone matrix. The diminutive size of these channels results in high shear stress levels, which are crucial for cellular function.^[^ ^124^ ^]^ Physiological levels of FSS have been shown to promote the migration and osteogenic differentiation of mesenchymal stem cells (MSCs).^[^ ^125^, ^126^, ^127^ ^]^ Furthermore, recent studies have revealed that FSS can increase the expression and activity of hyaluronic acid synthase (HAS), leading to the formation of microvilli‐like protrusions on the cell surface, thereby facilitating MSC adhesion.

Key factors influencing the impact of FSS on osteogenesis include its magnitude, periodicity, and frequency. Limraksasin et al. identified 0.5 Pa as the optimal shear force for inducing osteogenic differentiation in mouse‐induced pluripotent stem cells (iPSCs).^[^ ^54^ ^]^ Similarly, when combined with a nanoscale‐organized environment, an FSS of 1.4 Pa induced morphological changes in osteoblasts and significant cellular rearrangement, though the specific signaling pathways involved remained unclear.^[^ ^128^ ^]^ In 2D systems, short‐term FSS (2 Pa at 2 Hz) generated by parallel plate flow chambers has been shown to promote the expression of Cox‐2, OPN, and Runx‐2 during early osteogenesis, while prolonged exposure enhances collagen production and matrix formation.^[^ ^129^ ^]^ In 3D environments, MSCs demonstrate heightened sensitivity to FSS. For instance, naturally derived hydroxyapatite scaffolds (B‐HA) exposed to FSS (2.2–3.5 Pa) facilitated cell elongation in the direction of flow and early osteoblast differentiation.^[^ ^130^ ^]^ Likewise, Vetsch et al. reported that high flow rates (0.061 ms^−1^) and shear forces (0.55–24 MPa) in 3D silk fibroin scaffolds significantly enhanced hMSC osteogenic differentiation. Excessive shear stress, however, may induce MSC apoptosis.^[^ ^56^ ^]^ Notably, intermittent FSS has been shown to outperform continuous FSS in promoting osteogenic differentiation on polylactic‐co‐glycolic acid (PLGA) 3D scaffolds.^[^ ^131^ ^]^

**Figure 4:**
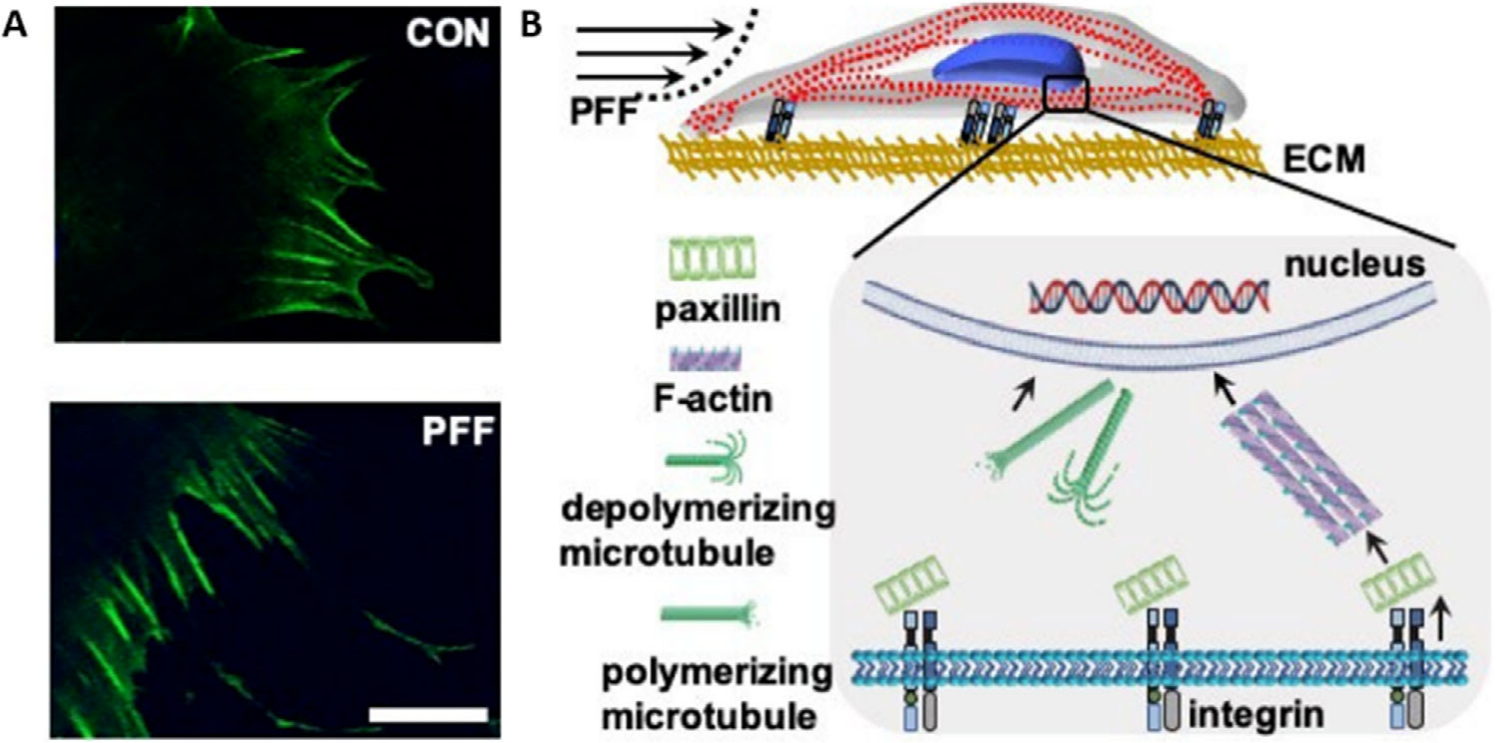
Effect of 1 h PFF on F‐actin distribution at the cell boundary, and schematic summary of the changes induced by PFF in MC3T3‐E1 osteoblasts. A) F‐actin was visualized using phalloidin. Upper image: control cell. Lower image: PFF‐treated cell. Bar = 25 µm. B) PFF was sensed through integrins, which formed clusters of α and β‐integrins. Paxillin changed from granular to short rods. F‐actin was neatly arranged, and F‐actin fluorescence intensity was increased by PFF. The morphology and volume of the cell body and nucleus changed in response to PFF, which might result in changes cell function. CON, control; PFF, pulsating fluid flow. Reproduced under the terms of the CC‐BY‐NC Creative Commons Attribution NonCommercial License 4.0 (https://creativecommons.org/licenses/by‐nc/4.0/).^[^ ^144^ ^]^ Copyright 2020, The authors, published by MDPI.

Mechanotransduction processes initiated by FSS include the rapid production of nitric oxide (NO) and prostaglandin E2 (PGE2), alongside modulation of canonical Wnt signaling.^[^ ^53^, ^132^, ^133^, ^134^ ^]^ Bioreactor‐generated FSS activates pathways such as BMP‐2, RhoA/Rock, Kif3a, ERK1/2, FAK, and Wnt, facilitated by mechanosensors including integrins, primary cilia, calcium channels, TRPV4, and Piezo1.^[^ ^53^, ^56^, ^57^, ^135^, ^136^, ^137^, ^138^, ^139^, ^140^, ^141^, ^142^, ^143^ ^]^ For example, FSS reorganizes the cytoskeleton, modulates α‐tubulin orientation, and increases actin concentration around the nucleus, as observed in MC3T3‐E1 osteoblasts under pulsating fluid flow (PFF) (**Figure** **4**).^[^ ^144^ ^]^ FSS‐induced morphological changes involve extracellular collagen and focal adhesions, which play direct roles in osteogenic responses.^[^ ^145^ ^]^

#### 3.1.6 Viscoelasticity

Viscoelasticity, a time‐dependent mechanical property, combines features of both elastic solids and viscous liquids. It is characterized by two key behaviors: stress relaxation, the gradual reduction of stress under constant strain, and creep, the progressive deformation of a material under a sustained load, often accompanied by hysteresis and nonlinear elasticity, such as strain hardening.^[^ ^58^, ^146^, ^147^ ^]^ This property is critical in biomaterials as it closely replicates the natural extracellular matrix (ECM). Beyond matrix stiffness, degradation, or adhesion ligand density, the dynamic mechanical properties of the ECM—specifically viscoelasticity—profoundly affect the behavior and differentiation of mesenchymal stem cells (MSCs).^[^ ^148^, ^149^ ^]^ Recently, increasing attention has been directed toward understanding how the viscoelasticity of bone ECM influences cellular behaviors, including adhesion, migration, proliferation, differentiation, and mineralization.

**Figure 5:**
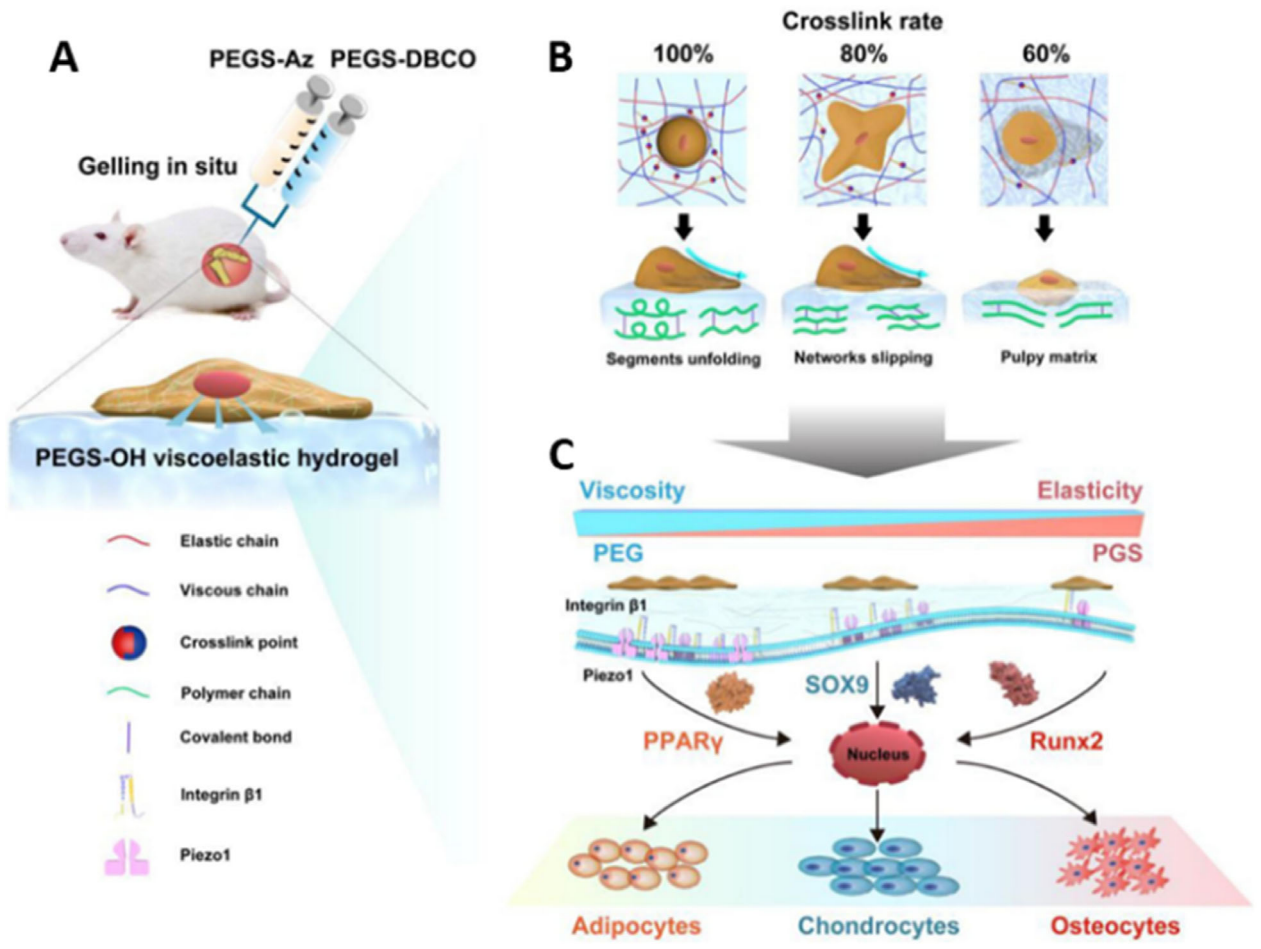
A) Injectable PEGS‐OH hydrogels form via a click reaction under physiological conditions, then BMSCs adhesion and differentiation occur on the viscoelastic PEGS‐OH hydrogel. B) The cross‐link rate prominently influences adhesion and differentiation of BMSCs. On the 100% crosslinked hydrogel, the relaxation of cellular stress occurs just by flexible segments unfolding, and on the 60% cross‐linked hydrogel, BMSCs are caught in a pulpy matrix. Only on the 80% crosslinked hydrogel, partly incomplete network slipping exerts better adhesion and differentiation effects on BMSCs. C) The content of PEG also regulates BMSCs differentiation. Viscosity provided by flexible segments (PEG) is beneficial to BMSCs, exerting better adhesion and differentiation into soft tissue. The promotion of BMSCs’ adhesion and differentiation was based on Piezo1 signal pathway. Reproduced with permission.^[^ ^152^ ^]^ Copyright 2021, Elsevier.

Recent studies reveal that bone marrow stem cells (BMSCs) can sense the viscoelasticity of materials or the extracellular matrix (ECM) through TRPV4 ion channels.^[^ ^150^, ^151^ ^]^ Notably, experiments have demonstrated that 80% cross‐linked polyethylene glycol diacrylate (PEGS‐OH) hydrogels exhibit tailored viscoelastic properties that enhance BMSC adhesion and differentiation, a process mediated by the mechanosensitive channel Piezo1 (**Figure** **5**).^[^ ^152^ ^]^ Stress relaxation, a key feature of viscoelasticity, significantly influences cell behavior. MSCs cultured in 3D gels with varying stress relaxation times exhibit distinct responses. Faster stress‐relaxing sodium alginate hydrogels (≈50 s) accelerate the cell cycle, promoting cell diffusion, proliferation, and osteogenic differentiation.^[^ ^62^ ^]^ Moreover, sodium alginate hydrogels coupled with RGD peptides, possessing an optimal modulus (≥ ≈17 kPa) and relatively rapid stress relaxation (60–300 s), effectively enhance cell spreading, osteogenic differentiation, and MSC spheroid fusion.^[^ ^58^, ^59^ ^]^ These effects are driven by the mechanical clustering of adhesion ligands, actomyosin contractility, and subsequent ECM remodeling.^[^ ^58^, ^62^, ^153^ ^]^ MSCs cultured in viscoelastic matrices with rapid stress relaxation demonstrate robust migration, strong cell affinity, and enhanced osteogenic differentiation in vitro.^[^ ^59^, ^153^, ^154^ ^]^

The extracellular viscoelastic properties of sodium alginate hydrogels have been shown to induce osteogenic differentiation through a cascade of mechanisms, including integrin clustering, interaction with RGD ligands, actomyosin contractility, YAP nuclear translocation, and activation of the PI3K/Akt signaling pathway.^[^ ^58^, ^62^ ^]^ Furthermore, chitin whisker‐assembled poly(l‐lactide) scaffolds can enhance both osteogenesis and angiogenesis by activating the HIF‐1α signaling pathway.^[^ ^153^ ^]^

#### 3.1.7 Hydrostatic Pressure

Hydrostatic pressure (HP) plays a pivotal role in bone tissue regeneration.^[^ ^155^ ^]^ In the context of bone tissue engineering, it has been estimated that osteocytes within the canaliculi‐lacunae network of load‐bearing bone experience pressures of ≈0.27 MPa. This underscores the importance of HP in replicating the physiological conditions necessary for in vivo bone formation.^[^ ^156^ ^]^ HP has attracted significant attention as a key mechanical stimulus capable of promoting 3D cell migration, cell cycle progression, and angiogenic sprouting, all of which are vital processes in bone regeneration.^[^ ^157^ ^]^ However, a study by Henstock revealed that applying a constant pressure of 279 kPa did not enhance bone mineralization. This finding highlights the necessity of dynamic force application as a critical factor for stimulating bone growth effectively.^[^ ^158^ ^]^ These insights underscore the importance of dynamic HP as a strategy to mimic natural mechanical cues in bone tissue engineering, offering promising potential for advancing regenerative techniques.

Research has demonstrated significant differences in cellular behavior between 2D and 3D environments. In 2D cultures, cells experience primarily unidirectional physical forces, which fail to adequately replicate the multidirectional mechanical loads encountered under physiological conditions.^[^ ^159^ ^]^ This discrepancy underscores the limitations of 2D models in mimicking the natural cellular microenvironment. In contrast, the dimensionality of 3D models provides a more physiologically relevant context, enabling a more accurate representation of how mechanical stimuli influence cellular responses. To better understand the complex mechanotransduction processes, it is essential to explore cellular behavior under hydrostatic pressure in controlled 3D environments. Further research in this area holds the potential to advance our knowledge of cell‐matrix interactions and improve the design of biomimetic systems that replicate the dynamic mechanical conditions of natural tissues.

**Figure 6:**
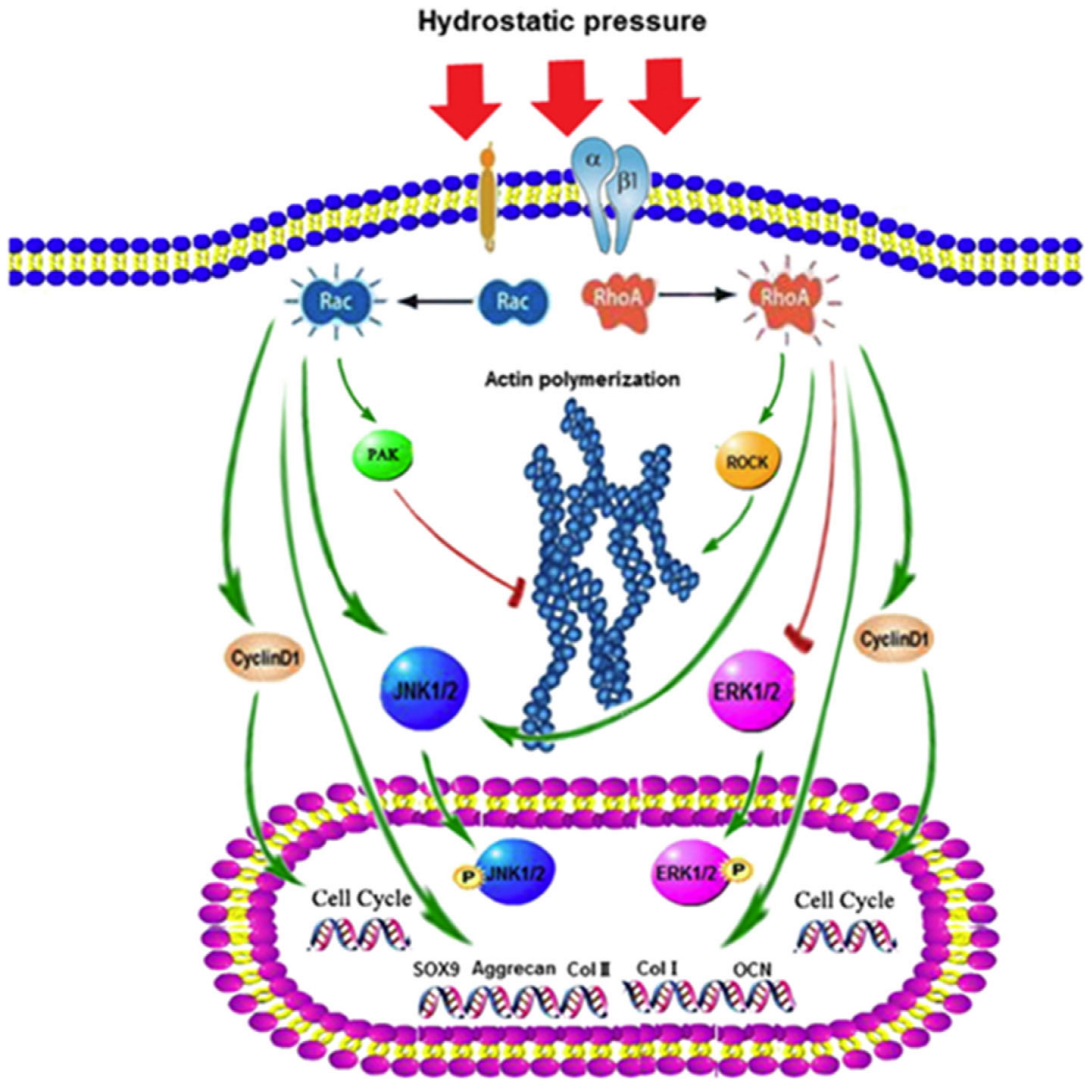
Schematic diagram shows the details of RhoA and Rac1 involved in the mechanotransduction, proliferation, and osteogenic/chondrogenic differentiation of BMSCs. A) Hydrostatic pressure regulates cell cycle initiation through both the RhoA/Rock and the Rac1 signaling pathways. At the same time, the mechanical stimulation promoted cytoskeletal assembly in BMSCs through the up‐regulation of RhoA/ROCK activities, and activation of the JNK1/2 pathway by down‐regulation of Rac1 activity. B) Hydrostatic pressure could also enhance the expression of marker genes for early osteogenic differentiation through the up‐regulation of RhoA activation or enhance the expression of chondrogenic marker genes in BMSCs during chondrogenic differentiation via the up‐regulation of Rac1 activity. Reproduced with permission.^[^ ^64^ ^]^ Copyright 2015, Elsevier.

Research demonstrates that a bioreactor generating cyclic hydrostatic pressure (HP) in the range of 0–279 kPa at a frequency of 1 Hz for 60 min significantly enhances calcium deposition, a result attributed to HP‐induced angiogenic differentiation of rMSCs and subsequent capillary formation in the CAM model.^[^ ^160^ ^]^ HP has also been shown to alter ion channel conformation and regulate transmembrane ion transport, thereby influencing various pathophysiological processes.^[^ ^161^ ^]^ For instance, Piezo1 can respond to HP by activating the ERK1/2 and p38 MAPK signaling pathways, promoting the expression of bone morphogenetic protein 2 (BMP2) and affecting the phenotype of mesenchymal stem cells (MSCs).^[^ ^63^ ^]^ Further studies by Liu et al. revealed that the ERK pathway plays an active, though non‐essential, role in early bone differentiation mediated by mechanotransduction, whereas the p38 MAPK pathway is not involved in this process.^[^ ^162^ ^]^ Additionally, the roles of RhoA and Rac1 are critical in HP‐mediated MSC proliferation and differentiation. These pathways also regulate the assembly of F‐actin stress fibers and JNK1/2 activation in BMSCs, highlighting their influence on cellular behavior (**Figure** **6**).^[^ ^64^ ^]^ Notably, cyclic HP has been identified as a powerful stimulus for cytoskeletal remodeling, which enhances the osteogenic response of MSCs. The intermediate filament (IF) network undergoes disassembly and reorganization under HP, with IF bundles relocating centripetally toward the perinuclear region—an essential process for load‐induced osteogenesis.^[^ ^163^ ^]^ Dynamic application of HP preserves the mechanosensitivity of MSCs, ensuring their responsiveness to physical stimuli.^[^ ^159^ ^]^

In conclusion, periodic or intermittent HP within the range of 10–300 kPa effectively induces osteogenesis in MSCs, making it a promising approach for enhancing bone regeneration.^[^ ^164^ ^]^

### 3.2 Biochemical Signals

Stem cells engage in direct interactions with the membrane proteins of supporting cells within their niche. These supporting cells anchor stem cells via surface adhesion proteins, positioning them in proximity to self‐renewal signals emitted by the niche environment.^[^ ^165^ ^]^ Leveraging biomaterials modified with cell‐contact‐mimicking domains has emerged as a promising strategy to modulate regenerative niches, facilitating the targeted differentiation of stem cells, including pluripotent and mesenchymal stem cells.

The development of biomaterials that replicate intercellular interactions remains in its early stages within the field of bone regeneration. Zhu et al. designed a hydrogel modified with methacrylated hyaluronic acid, incorporating N‐cadherin and integrin‐binding domains to create an “in situ” microenvironment for MSCs.^[^ ^166^ ^]^ Similarly, Huang et al. biofunctionalized the internal micropillars of 3D‐SCµN with fibronectin (FN) and E‐cadherin (E‐Cad) ligands to mimic cell‐matrix and cell‐cell interactions, respectively. Their experiments revealed that biophysical niche signals can control the direction of cell division independently of cell shape.^[^ ^167^ ^]^ These findings highlight the potential for biomimetic materials that integrate ECM and intercellular interaction cues, paving the way for advanced biomaterials that better elucidate and leverage the dynamics of stem cell niches.

Soluble molecules play a pivotal role in guiding stem cell fate and, due to their accessibility for study, have become primary targets for regulating stem cell behavior in vitro. Growth factors, as reviewed by Wang et al., are extensively studied for their impact on stem cell dynamics.^[^ ^168^ ^]^ Beyond secreted proteins, small metabolic molecules also serve as essential cues within the stem cell niche.^[^ ^169^, ^170^ ^]^ Peptides are widely utilized in bone regeneration studies, with diverse peptides demonstrating varying effects. For instance, BMP‐2‐mimicking peptides conjugated to alginate‐maleimide microcapsules have been shown to enhance osteogenic differentiation.^[^ ^171^ ^]^ Recently, modularly designed biomimetic ultra‐short peptide nanofiber hydrogels have been employed to construct osteogenic immune microenvironments.^[^ ^172^ ^]^ Aptamers, highly specific nucleic acid sequences, are increasingly used to target specific cells in bone regeneration. For example, aptamers have been developed to selectively bind human pluripotent stem cells, while functionalized scaffolds coupled with aptamers have successfully stimulated the directional differentiation of mesenchymal stem cells and supported tissue formation.^[^ ^173^, ^174^ ^]^ Liang et al. identified an osteoblast‐specific aptamer, CH6, which has been incorporated into extracellular vesicle‐based platforms to promote mineralization and bone regeneration in vivo.^[^ ^175^, ^176^ ^]^ While growth factors are effective, their short shelf life, high production costs, and instability present challenges compared to peptides and aptamers. Peptides offer low immunogenicity and ease of modification, although their specificity is lower than that of aptamers. Aptamers, with their high specificity, low immunogenicity, and cost‐effective production, are poised to become powerful tools for targeted interventions in biomimetic materials.^[^ ^177^ ^]^

## 4 Biomimetic Nanomaterials for Designing Stem Cell Niches

The interplay of mechanical and biochemical cues within the nanoscale environment is pivotal in determining stem cell fate. It is increasingly recognized that the extracellular matrix (ECM) is far more than an inert structural scaffold—it serves as a dynamic information center, regulating cellular behavior through a myriad of spatiotemporal signals. Simulating and integrating these complex cues into next‐generation ECM‐mimicking scaffolds has the potential to revolutionize tissue repair and regeneration. This section explores recent advancements in biomimetic nanomaterials, focusing on their application in model stem cell niche environments and tissue engineering.^[^ ^178^ ^]^

### 4.1 Nanomaterials

Scaffolds for bone tissue engineering (BTE) must exhibit key biological properties, including biocompatibility, bioactivity, and biodegradability, to interact effectively with surrounding cells and tissues, thereby promoting bone regeneration.^[^ ^179^ ^]^ Biocompatibility ensures that scaffold components are non‐toxic and support cell survival, growth, and functionality without adverse effects.^[^ ^180^ ^]^ Bioactivity refers to the scaffold's capacity to stimulate osteogenesis through osteoinductivity, osteoconductivity, and osseointegration.^[^ ^181^, ^182^, ^183^, ^184^ ^]^ Biodegradability allows the scaffold's degradation rate to synchronize with new bone formation, facilitating efficient bone repair.^[^ ^185^, ^186^, ^187^ ^]^ The following subsections examine the roles of inorganic fillers and organic matrices in achieving these goals.

#### 4.1.1 Inorganic Fillers

Nanomaterials are fundamental components of nanotechnology, defined by at least one dimension measuring less than 100 nm. These materials exhibit diverse shapes, including nanoparticles, nanowires, and nanosheets, which can be characterized based on their dimensions.

0D nanomaterials encompass nanoparticles, such as mineral particles and carbon‐based nanomaterials.^[^ ^168^, ^188^ ^]^ Due to their high surface‐to‐volume ratio, these biomaterials possess exceptional properties, including remarkable mechanical strength, thermal conductivity, electrical performance, and surface reactivity, which collectively enable them to regulate cell behavior and promote bone mineralization.

1D nanomaterials include nanowires and nanotubes,^[^ ^189^ ^]^ such as carbon nanotubes (CNTs), titanium dioxide nanotubes (TNTs), halloysite nanotubes (HNTs), hydroxyapatite nanotubes (HANTs),^[^ ^190^ ^]^ and hydroxyapatite nanowires. Other examples are 1D silicate nanomaterials, silica nanofibers, bioglass nanofibers, yttrium‐stabilized zirconia nanofibers, and β‐tricalcium phosphate nanofibers.^[^ ^191^ ^]^ These biomaterials exhibit unique nanoscale morphologies and a high aspect ratio, which significantly influence cell behavior and calcium biomineralization. Parameters such as the diameter and alignment of nanowires and nanotubes are particularly critical for mesenchymal stem cell (MSC) differentiation, necessitating further research.

2D nanomaterials include nanosheets, such as graphene, graphene oxide, black phosphorus nanosheets, 2D nanoclays, layered double hydroxide nanosheets (LDHs), transition metal carbides, nitrides, and carbonitrides (MXenes),^[^ ^192^ ^]^ as well as transition metal dichalcogenides (TMDs), transition metal oxides (TMOs), and two‐dimensional metal‐organic frameworks (MOFs).^[^ ^193^, ^194^ ^]^

The large aspect ratio of 2D nanomaterials offers significant potential for modifying complex surface characteristics, such as surface chemistry and charge, of biomaterials. These modifications can substantially influence cellular behavior and fate.^[^ ^189^ ^]^ Furthermore, their nanoscale dimensions enhance their physicochemical properties, endowing them with unique characteristics that make them highly effective in bone tissue engineering applications.^[^ ^195^ ^]^ Owing to their small size and high chemical reactivity, nanomaterials exhibit enhanced cellular uptake and increased potential for interactions with biomolecules and tissues.^[^ ^196^ ^]^ These nanomaterials are capable of directly damaging cells and organelles, inducing unregulated cellular signaling, releasing toxic ions, and generating ROS, all of which can lead to DNA damage.^[^ ^197^ ^]^ To mitigate these adverse effects, strategies such as organic molecule modification (e.g., PEG), polysaccharide coating, functional polymer encapsulation, and hydrophilic surface modification can be employed to reduce toxicity.^[^ ^198^, ^199^ ^]^

For additional details on the specific properties and applications of these nanomaterials, refer to **Table** **2**.

**Table 2:** Types of nanomaterials with different dimensions.

Dimensionality	Classification	Nanomaterials	Refs.
Zero dimension	Mineral particle Carbon nanomaterial	Nano‐hydroxyapatite	[200, 201]
		Iron oxide nanoparticle	[202, 203]
		Zinc oxide nanoparticle	[204, 205]
		Alumina nanoparticle	[206]
		Magnesium oxide nanoparticle	[207]
		Silica nanoparticle	[208]
		Nanodiamond (ND)	[209]
		Carbon dot(CD)	[210, 211]
One dimension Two dimension	Nanowire Nanotube Nanosheet	Hydroxyapatite nanowire	[212]
		Silica nanofiber	[213, 214]
		Bioglass nanofiber	[215, 216]
		Zirconia nanofiber	[217]
		Beta‐tricalcium phosphate nanofiber	[218]
		Carbon nanotube	[219, 220]
		Titanium oxide nanotube	[221, 222]
		Allostone nanotube	[223, 224]
		Hydroxyapatite nanotube	[225]
		Graphene	[226]
		Graphene oxide	[227, 228]
		Black phosphorus nanosheet	[229, 230]
		Clay nanosheet	[231]
		Layered double hydroxide	[232, 233]
		MXene	[234, 235]

#### 4.1.2 Organic Matrices

Hydrogels represent one of the most versatile and widely used biomaterials, owing to their compatibility with natural tissues and the extracellular matrix (ECM).^[^ ^236^ ^]^ Their porous structure facilitates the exchange of nutrients and metabolic waste, thereby enhancing cell viability. For tissue engineering applications, hydrogels can be synthesized from a variety of natural and synthetic polymers.^[^ ^237^ ^]^

Natural biopolymers, such as chitosan,^[^ ^238^, ^239^, ^240^, ^241^ ^]^ sodium alginate,^[^ ^242^, ^243^, ^244^, ^245^ ^]^ collagen,^[^ ^246^, ^247^, ^248^, ^249^ ^]^ silk fibroin,^[^ ^250^, ^251^, ^252^ ^]^ hyaluronic acid,^[^ ^253^, ^254^, ^255^ ^]^ and gelatin,^[^ ^256^, ^257^, ^258^ ^]^ offer several advantages, including low toxicity, high biocompatibility, intrinsic biodegradability, strong bioactivity, and excellent cell affinity. These attributes make them highly suitable for use in bone tissue engineering.

Synthetic biopolymers, including polycaprolactone (PCL),^[^ ^259^, ^260^, ^261^ ^]^ polyethylene glycol (PEG),^[^ ^262^, ^263^ ^]^ polylactic‐co‐glycolic acid (PLGA),^[^ ^264^, ^265^ ^]^ polyvinyl alcohol (PVA),^[^ ^266^, ^267^ ^]^ and polylactic acid (PLA),^[^ ^268^, ^269^ ^]^ provide additional benefits such as superior plasticity and reproducibility. These polymers allow precise manipulation of hydrogel properties during polymerization and subsequent modifications, such as cross‐linking and functionalization. As a result, synthetic hydrogels can be tailored in terms of block architecture, viscosity, mechanical properties, and biodegradability to meet specific requirements.^[^ ^191^ ^]^

Additionally, hydrogels are often combined with 0D, 1D, and 2D biomaterials to form 3D scaffolds, harnessing the outstanding biological effects of these materials for enhanced tissue regeneration.

### 4.2 Nanotechnology

Nanocomposites have become a cornerstone in bone tissue engineering (BTE),^[^ ^270^ ^]^ largely due to the hierarchical structure of bone, which comprises hydroxyapatite (HA) nanocrystals embedded in a collagen fiber matrix.^[^ ^271^ ^]^ Bone cells naturally interact with nanostructured materials, leveraging the rough surfaces and pore sizes (≈2100 nm) that are inherent to their biological environment.^[^ ^272^ ^]^

Recent years have witnessed significant advancements in the application of nanotechnology to BTE. Nanotechnology offers innovative solutions to improve the physical, mechanical, and biochemical properties of scaffolds. For instance, nanostructured materials can enhance cellular adhesion, promote osteogenesis, and provide tailored degradation rates, making them invaluable for creating biomimetic scaffolds.^[^ ^195^, ^273^ ^]^

#### 4.2.1 Electrospinning

Electrospinning is a versatile and efficient technique for producing nanofibers from a wide range of materials. This method enhances the structural, porosity, surface, and orientation characteristics of nanofibers.^[^ ^274^ ^]^ During electrospinning, a viscoelastic polymer solution is extruded through a stainless‐steel needle under high voltage, resulting in the formation of nanofibers.^[^ ^274^, ^275^ ^]^ Its versatility lies in the ability to process various polymer materials, including collagen (Col), chitosan (CTS), cellulose, hydroxyapatite (HA), polylactic acid (PLA), polycarbonate (PC), and PCL/PLA blends, to mimic the hierarchical structure of the extracellular matrix (ECM) in 3D environments. These structures are critical for regulating cell behavior in bone regeneration, as they promote cell infiltration, viability, and functionality.^[^ ^99^, ^274^, ^276^ ^]^

The physicochemical properties of electrospun scaffolds—such as fiber alignment, nanoscale morphology, porosity, mechanical strength, and biodegradability—can be precisely tailored by adjusting the electrospinning parameters and employing integrated techniques.^[^ ^277^, ^278^, ^279^ ^]^ Optimizing these properties is essential, as they significantly influence cellular behaviors and biological functions. For example, highly aligned fibers support the directional growth of seeded cells through contact guidance, while scaffold stiffness facilitates the differentiation of mesenchymal stem cells (MSCs) into specific lineages.^[^ ^280^ ^]^ Chen et al. developed electrospun polycaprolactone (PCL) nanofibers with a hierarchical structure and pre‐designed 3D aligned nanoscale morphology. These biomaterials guided bone marrow stem cells (BMSCs) to migrate from peripheral areas toward the center, promoting regeneration. The regenerated bone exhibited an organic phase with aligned, densely packed structures, and an inorganic phase with uniformly distributed minerals, smaller pores, and consistent stress distribution under compression.^[^ ^281^ ^]^ Similarly, Xu et al. designed a PCL‐based 3D nanofiber scaffold with stratified structural pores created through thermal‐induced self‐aggregation and freeze‐drying. This scaffold featured large pores (≈300 µm), high porosity (96.4%), and soft, elastic properties, making it highly analogous to natural ECM and ideal for supporting cell functionality and tissue formation.^[^ ^282^ ^]^

The functionality of electrospun nanofibers can be further enhanced by modifying their structure or incorporating bioactive molecules and nanoparticles, such as nano‐hydroxyapatite (nHA),^[^ ^283^ ^]^ bone morphogenetic protein‐2 (BMP‐2),^[^ ^284^ ^]^ and graphene oxide (GO).^[^ ^285^ ^]^ These additions improve osteogenic outcomes by enhancing surface roughness and bioactivity. For instance, nHA incorporation creates a nanostructured surface that preferentially promotes cell adhesion.^[^ ^286^ ^]^ He et al. fabricated mineralized nanofiber scaffolds with layered structures using template‐assisted electrospinning and mineral deposition, combining anisotropic, and isotropic fiber morphologies with mineralized particles for enhanced osteogenesis.^[^ ^287^ ^]^ Meanwhile, Long et al. developed silk fibroin nanofiber aerogels (SNFAs) with a layered structure, parallel‐aligned channels, and adjustable mechanical properties. The SNFAs mimic the ECM‘s fibrous architecture and possess a Young‘s modulus adjustable from 7 to 88 kPa, closely matching the mechanical properties of bone tissue.^[^ ^288^ ^]^

Despite current challenges, electrospinning holds immense potential for producing nanofiber scaffolds with controllable compositions and structures. Its adaptability allows researchers from various disciplines to design novel scaffolds with biomimetic nanoscale characteristics tailored for tissue engineering. The continued advancement of electrospinning technology promises to drive further innovation in the design and application of biomimetic scaffolds, significantly contributing to the evolving field of regenerative medicine.

#### 4.2.2 3D Printing

3D printing is a groundbreaking manufacturing process that constructs objects layer by layer from 3D computer models, enabling the creation of complex geometries with high precision.^[^ ^289^ ^]^ Compared to traditional molding techniques, 3D printing offers several distinct advantages, including exceptional flexibility in fabricating intricate structures, rapid prototyping of multi‐scale layered pore features, and the ability to utilize diverse biomaterials to replicate physiological microenvironments akin to natural bone.^[^ ^290^, ^291^ ^]^ However, the resolution of current 3D printing technologies is constrained to the microscale, limiting their ability to produce biomimetic functional scaffolds with nanoscale hierarchical structures, particularly in terms of surface nanomorphology.^[^ ^7^ ^]^ Integrating advanced nanotechnology with 3D printing holds immense potential for overcoming these limitations, paving the way for innovative designs in bone tissue engineering scaffolds.

Numerous researchers have explored incorporating nanostructures—such as nanoparticles, nanofibers, and 2D nanomaterials—into polymer matrices to tailor the physical, mechanical, and biochemical properties of 3D‐printed scaffolds, effectively mimicking the nanocomposite structure of natural bone.^[^ ^253^, ^292^, ^293^ ^]^ Liang et al. introduced an “organic‐inorganic assembly” strategy to fabricate SF‐derived bone scaffolds with biomimetic mechanical properties, 3D structures, and enhanced osteogenic performance. Through chemical modification, SF was processed via digital light processing (DLP) 3D printing, resulting in hydrogel scaffolds with biomimetic and customizable 3D structures and a compressive modulus increased by 47‐fold (from 0.05 MPa to 2.33 MPa).^[^ ^294^ ^]^ Similarly, Jiang et al. developed a high‐precision bioinspired bone scaffold made of methacrylated polycaprolactone (PCLMA) using DLP‐based 3D printing technology. By employing a perfusion device, they uniformly and stably integrated adipose‐derived stem cell‐engineered nanovesicles (ADSC‐ENs) onto the scaffold surface, creating a microenvironment optimized for tissue regeneration and the repair of long bone defects, leveraging both the scaffold's structural design and the biological functions of the nanovesicles.^[^ ^295^ ^]^

**Figure 7:**
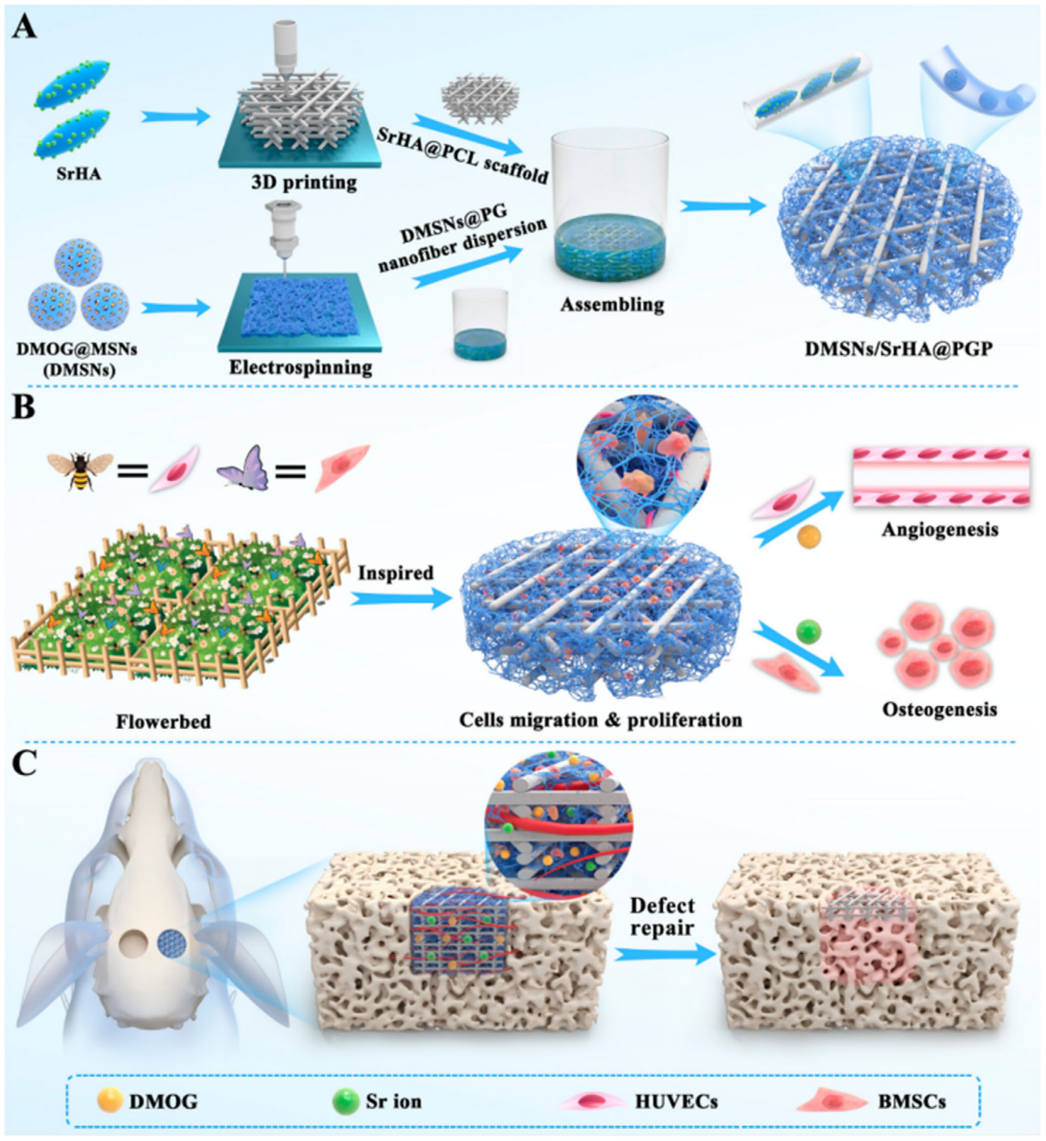
A) Schematic Illustration of the Fabrication of DMSNs/SrHA@PGP Scaffold to Co‐deliver Angiogenic Drug and Osteogenic Metal Ion via the Combination of 3D Printing and Electrospinning Techniques. B) Dual‐Factor Delivery Scaffold Provides the Favorable Extracellular Microenvironment for Cell Migration, Proliferation, and Differentiation as the Flowerbed Provides the Flowers to Attract the Bees and Butterflies. C) Combinational Delivery of DMOG and Sr Ions in DMSNs/SrHA@PGP Scaffold Could Synergistically Improve the Repair Efficacy of Bone Defects. Reproduced with permission.^[^ ^298^ ^]^ Copyright 2023, American Chemical Society.

Nanofibers offer remarkable potential in bone tissue engineering due to their ability to replicate the highly biomimetic structure of the extracellular matrix (ECM), which is crucial for emulating bone tissue. Liu et al. recently designed a mixed bilayer scaffold by integrating electrospinning and 3D printing techniques. The upper layer consists of a 2D heparin‐coupled PCL/GEL electrospun nanofiber membrane with ≈60% porosity and pore sizes of 500–600 µm, achieving a tensile strength of 10.22 MPa. The gelatin (GEL) component features RGD peptides, which enhance cell activation and proliferation via intrinsic cellular signaling pathways.^[^ ^296^ ^]^ To reinforce the upper membrane and prevent collapse, the lower layer comprises a 3D‐printed porous PCL/GEL/HAp scaffold with ≈59% porosity, an average pore size of 600 µm, and a tensile strength of 13.86 MPa, showcasing improved mechanical properties and structural porosity.^[^ ^297^ ^]^ The biomimetic approach has garnered significant research attention. Zhou et al. developed a biomimetic scaffold with a porous structure, impressive compressive strength, and tailored degradation properties by incorporating short nanofibers loaded with dimethyl oxalyl glycine (DMOG) into 3D‐printed strontium hydroxyapatite/polycaprolactone (SrHA@PCL) scaffolds. This scaffold demonstrated a mechanical stress of 1.87 ± 0.13 MPa and a compressive modulus of 29.85 ± 4.75 MPa, values within the range of human trabecular bone (compressive strength: 0.9–7.4 MPa; compressive modulus: 20–640 MPa). The scaffold effectively accelerated endogenous regeneration and vascularization during bone healing (**Figure** **7**).^[^ ^298^ ^]^ Xiao et al. further advanced scaffold design by developing a novel biomimetic scaffold with active ion release. By combining template‐assisted electrospinning with advanced 3D printing technology, they created a layered porous design that preserves the high specific surface area of nanofiber membranes while closely mimicking the ECM.^[^ ^299^ ^]^

With the advent of 2D materials, biomaterials have achieved notable technological advancements, particularly in the development of biomimetic nanoscaffolds for bone tissue regeneration. These 2D nanomaterials can seamlessly integrate with organic matrices, enhancing the functionality of nanoscaffolds. Rashidi et al. engineered a scaffold with a heterogeneous structure using dual‐nozzle 3D printing technology. The inner layer features a poly(ε‐caprolactone) (PCL) scaffold embedded with synthetic hydroxyapatite (HA) and graphene oxide (GO) nanosheets (GO@HAs), while the outer layer comprises PCL and natural allograft HA (HA ink). Field‐emission scanning electron microscopy (FE‐SEM) of the 3D‐printed scaffolds revealed a well‐defined macroporous structure with fully interconnected pores. The incorporation of GO nanosheets prevents HA nanoparticle aggregation, significantly enhancing the scaffold's mechanical properties.^[^ ^300^ ^]^ Cai et al. designed a biomimetic 3D‐printed scaffold incorporating photosensitive black phosphorus “bone seeds” (BS). These “bone seeds” were electrostatically attached to the porous 3D polycaprolactone (PCL) scaffold via interactions between phosphate and amine groups, forming a PCL‐BS scaffold capable of actively capturing calcium ions. The scaffold demonstrated a compressive strength of 11.5 MPa and a Young's modulus of 56 MPa, effectively promoting the endogenous regeneration of bone defects.^[^ ^301^ ^]^

With the rapid advancement of 3D printing technology, researchers are increasingly focusing on leveraging these techniques to develop multilayer scaffolds with biomimetic structures. However, several challenges persist, including the construction of gradient scaffolds with improved interfacial bonding to better mimic the structural and functional gradients of natural tissues. Additionally, replicating the exact physical, mechanical, and biochemical properties of native bone tissue remains a significant hurdle. Despite these limitations, the integration of novel materials, advancements in 3D printing technology, and interdisciplinary scientific approaches offers promising prospects. Continued research in bone tissue engineering scaffolds is expected to pave the way for the effective regeneration of bone defects.

#### 4.2.3 Nanopatterning

**Figure 8:**
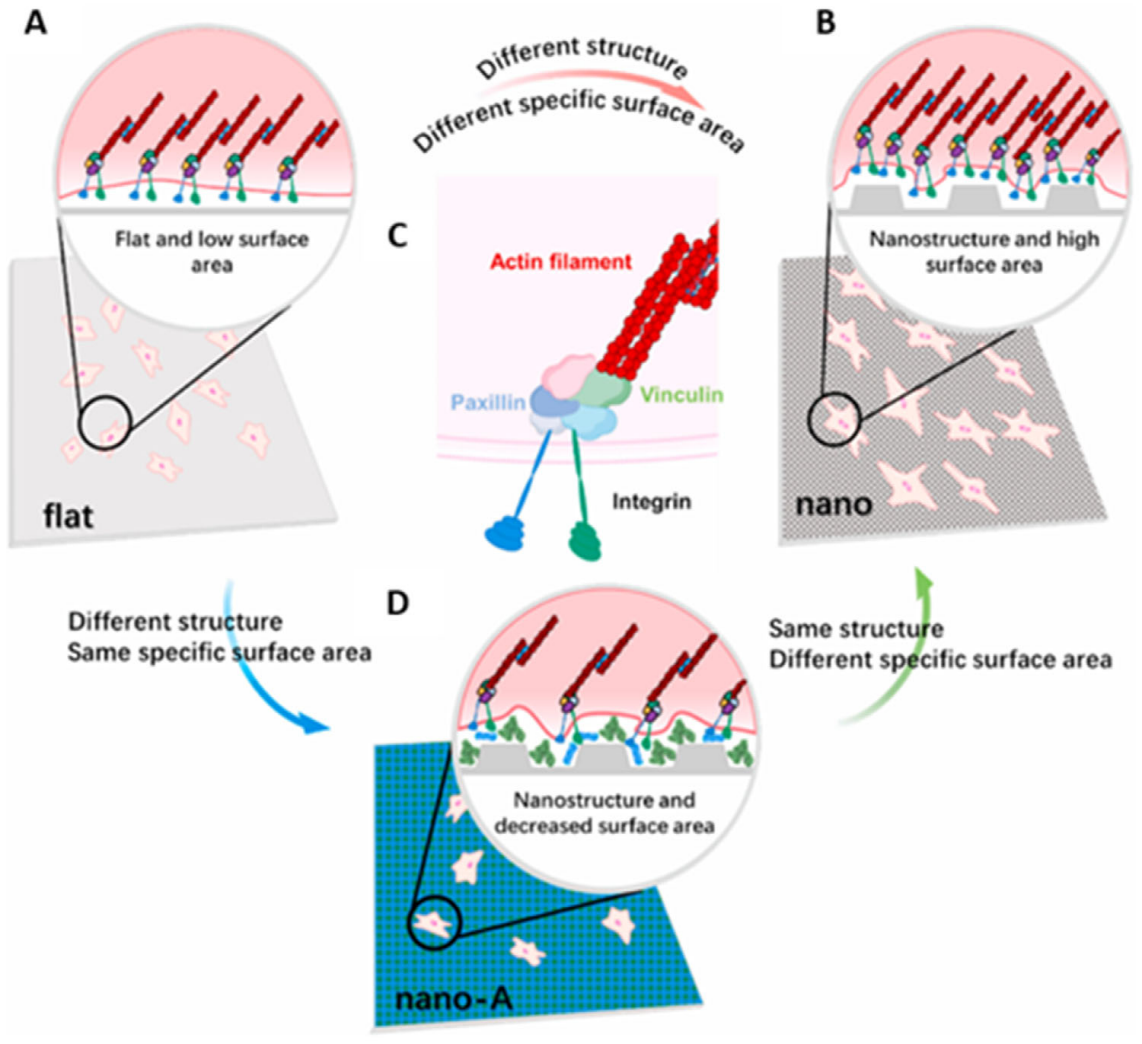
Schematic summary of cell adhesion and polarization on the “flat”, “nano”, and “nano‐A” surfaces. A) Scheme of cell adhesion on the “flat” (flat and low specific surface area). Cells on the “flat” spread less and show a rounded morphology. Aligned filaments in BMSCs on the “flat” are not as abundant as they are on the “nano”. The activity of myosin decreased on the “flat”, indicating a lower intercellular force in the cells. B) Scheme of cell adhesion and polarization on the “nano” (nanostructure and high specific surface area). Cells on the “nano” spread more and exhibit a lower cell shape circularity index. Actin filaments are more abundant and well‐aligned in BMSCs on the “nano”. The myosin force pathway for cellular forces is prominently activated on the “nano”. C) Schematic illustration of surface nanopattern induces the formation and the alignment of the focal adhesion, which promotes the intracellular force. D) Scheme of cell adhesion on the “nano‐A” (nanostructure and the cell‐perceivable surface specific area is equivalent to the area of “flat”). Cells on the “nano‐A” spread less and present a rounded morphology with few and short protrusions, which is similar to cell morphology on the “flat” surface. The activity of myosin decreased on the “nano‐A”, indicating a lower intercellular force in the cells. Reproduced with permission.^[^ ^303^ ^]^ Copyright 2024, Elsevier.

Biochemical cues are among the most extensively explored and defining stimuli in bone tissue engineering. Their distribution and abundance in the natural extracellular matrix (ECM) are highly dependent on the target cellular responses and the specific location of the stem cell niche.^[^ ^302^ ^]^ Nanopatterning, by increasing the surface area of biomaterials, enhances the aggregation of adhesion proteins and increases the number of molecular clamps, thereby facilitating the efficient transmission of mechanical signals (**Figure** **8**).^[^ ^303^ ^]^ Techniques such as microcontact printing enable the spatial patterning of bioactive factors on synthetic surfaces, offering precise control over their distribution.^[^ ^304^, ^305^, ^306^, ^307^, ^308^, ^309^ ^]^ For instance, photolithography provides an effective and accessible method for generating protein patterns on a variety of substrates. Recent studies have shown that nanopatterns can modulate stem cell behavior and influence lineage specification and differentiation.^[^ ^310^ ^]^

Wang et al. utilized block copolymer micelle nanolithography to produce gold nanostructures on glass, subsequently functionalized with cell‐adhesive arginine‐glycine‐aspartate (RGD) ligands to create peptide micro/nanopatterns. Their findings revealed that the nanoscale spacing of RGD does not directly affect cell spreading but serves as a modulator of cell tension and stem cell differentiation.^[^ ^311^ ^]^ However, the relationship between nanopatterning and stem cell differentiation remains complex. Dalby et al. investigated the effects of nanoscale topologies on mesenchymal stem cell (MSC) differentiation using five different patterns on polymethyl methacrylate (PMMA) prepared via electron beam lithography (EBL), each with a rcenter‐to‐center spacing of 300 nm. Their results demonstrated that highly ordered nanoscale morphologies had minimal impact on cellular adhesion or osteoblastic differentiation. In contrast, random nanoscale topologies significantly enhanced osteogenic differentiation and led to more osteoblast‐like morphologies.^[^ ^97^ ^]^ Similarly, micro/nanopatterned polyethylene terephthalate (PET) surfaces with varying pattern sizes, coverage, and shapes showed that disordered bioactive patterns were more effective in promoting osteogenic differentiation than highly ordered structures. These findings suggest that disordered patterning strategies may offer a promising approach for directing MSC behavior in bone regeneration and tissue engineering.^[^ ^312^ ^]^

Despite advancements in bottom‐up and top‐down manufacturing techniques, replicating the precise nanoscale and microscale morphology and chemical composition of bone tissue remains challenging. Current methods often fall short of creating biomimetic surfaces capable of accurately controlling cell behavior.^[^ ^313^ ^]^ Thermal scanning probe lithography (tSPL) offers a promising solution, enabling the fabrication of complex quasi‐3D morphologies on polymer surfaces with lateral resolutions below 15 nm and depth resolutions under 2 nm.^[^ ^314^ ^]^ Bio‐tSPL, an adaptation of tSPL, allows for the patterning of complex nanoscale structures across large areas. Researchers have successfully replicated the intricate quasi‐3D morphology of bone tissue using biocompatible polymer photoresists at resolutions below 15 nm. This technique supports stem cell growth and proliferation while offering a biomimetic platform for testing and controlling cell behavior. Consequently, bio‐tSPL represents a breakthrough in nanofabrication, opening new avenues for understanding cell‐tissue microenvironment interactions and providing precise control over stem cell fate and tissue regeneration.^[^ ^315^ ^]^

#### 4.2.4 Self‐Assembly

Self‐assembly leverages the interaction of small, unrelated components to construct large and complex structures. With its inherent scalability, self‐assembly holds great promise for creating biomimetic 3D tissue structures. The process occurs through various mechanisms, including the minimization of surface tension, geometric and chemical recognition, and biological interactions.^[^ ^316^ ^]^ For example, self‐assembled gelatin structures loaded with mesenchymal stem cells (MSCs) have been successfully used to produce macroscopic tissue architectures.^[^ ^317^ ^]^

Enhanced cell‐cell and cell‐ECM interactions significantly contribute to the self‐assembly of niches. Beyond facilitating the formation of larger tissues, peptide self‐assembly can be finely regulated by modifying the molecular structure of components undergoing self‐assembly. This enables adjustments to the morphology of surfaces and scaffolds. For instance, Stupp's team developed patches with thread‐like textures that thermally decompose into aligned nanofiber arrays, effectively directing cell orientation in 3D environments.^[^ ^318^ ^]^ Liquid crystals (LC) are a classic example of nanoscale self‐assembled structures. Nanoscale LC structures are naturally present in human cortical bone, suggesting that nanoscale self‐assembly closely mimics the processes involved in bone formation.^[^ ^319^ ^]^ Zhao et al. prepared oriented halloysite nanotubes on solid substrates, demonstrating that human bone marrow stem cells (hBMSCs) aligned effectively along the clay nanotubes.^[^ ^320^ ^]^ Another study showed that self‐assembled hydroxyapatite (HA) particles organized themselves into highly aligned nanoscale arrangements, creating regions with anisotropic pore structures.^[^ ^321^ ^]^ Furthermore, layer‐by‐layer (LBL) self‐assembly techniques can generate dual concentration gradients of bioactive components on electrospun nanofibrous membranes. These graded fiber scaffolds significantly enhance the migration, proliferation, and differentiation of bone marrow stem cells (BMSCs) by combining biological signals with topographical cues.^[^ ^322^ ^]^ Liquid crystal structures can integrate seamlessly into the bone microenvironment, serving as effective guides for cellular fate determination. By influencing cell behavior, these biomimetic approaches offer innovative strategies for tissue engineering.^[^ ^319^ ^]^

Self‐assembled structures may closely approximate the structural processes of natural bone formation, allowing precise control over scaffold size, shape, and orientation. However, self‐assembly techniques face significant challenges, including the complexity of fabrication and difficulty scaling for clinical applications. Variations in scaffold performance due to inconsistent structural control present further obstacles. Additionally, the range of materials suitable for self‐assembly is limited, and no current self‐assembling material matches the mechanical strength of natural bone.

## 5 Simulating Natural Bone Structure for Bone Tissue Engineering

Bone tissue features two primary niches: the osteoblastic niche and the vascular niche. These niches house two key types of stem cells: hematopoietic stem cells (HSCs) and mesenchymal stem cells (MSCs). Both cell types reside in bone marrow and vascularized bone cavities, collaborating to maintain normal bone homeostasis and cellular generation.^[^ ^28^ ^]^ This section reviews the applications of biomimetic nanomaterial design in replicating natural bone structure for bone tissue engineering. The discussion focuses on constructing: The skeletal system, the vascular system, the bone immune microenvironment, and Combined systems. These strategies aim to replicate the structural and functional complexity of native bone tissue, offering promising avenues for enhancing bone regeneration and repair.

### 5.1 Building the Bionic Skeletal System

#### 5.1.1 Matching Hierarchies

##### Biomimetic Bone Membrane

Bone membranes are essential for bone regeneration due to their abundant blood supply and intrinsic osteogenic activity. Acting as a local source of growth factors for osteoblast recruitment and as a capillary system, tissue‐engineered bone membranes have gained increasing attention in recent years. These membranes aim to replicate the structure and function of natural bone membranes, enhancing bone defect repair. Biomimetic bone membranes not only provide an optimal environment for bone healing but also reduce the risk of recurrent inflammation and necrosis at injury sites.^[^ ^323^ ^]^ Currently, artificial bone membranes fall into three main categories: cell sheet‐based, decellularized scaffold‐based, and synthetic scaffold‐based membranes. These designs emulate natural osteogenesis and vascularization, aligning more closely with physiological bone repair processes and thus improving the efficiency of bone regeneration.^[^ ^324^ ^]^

**Figure 9:**
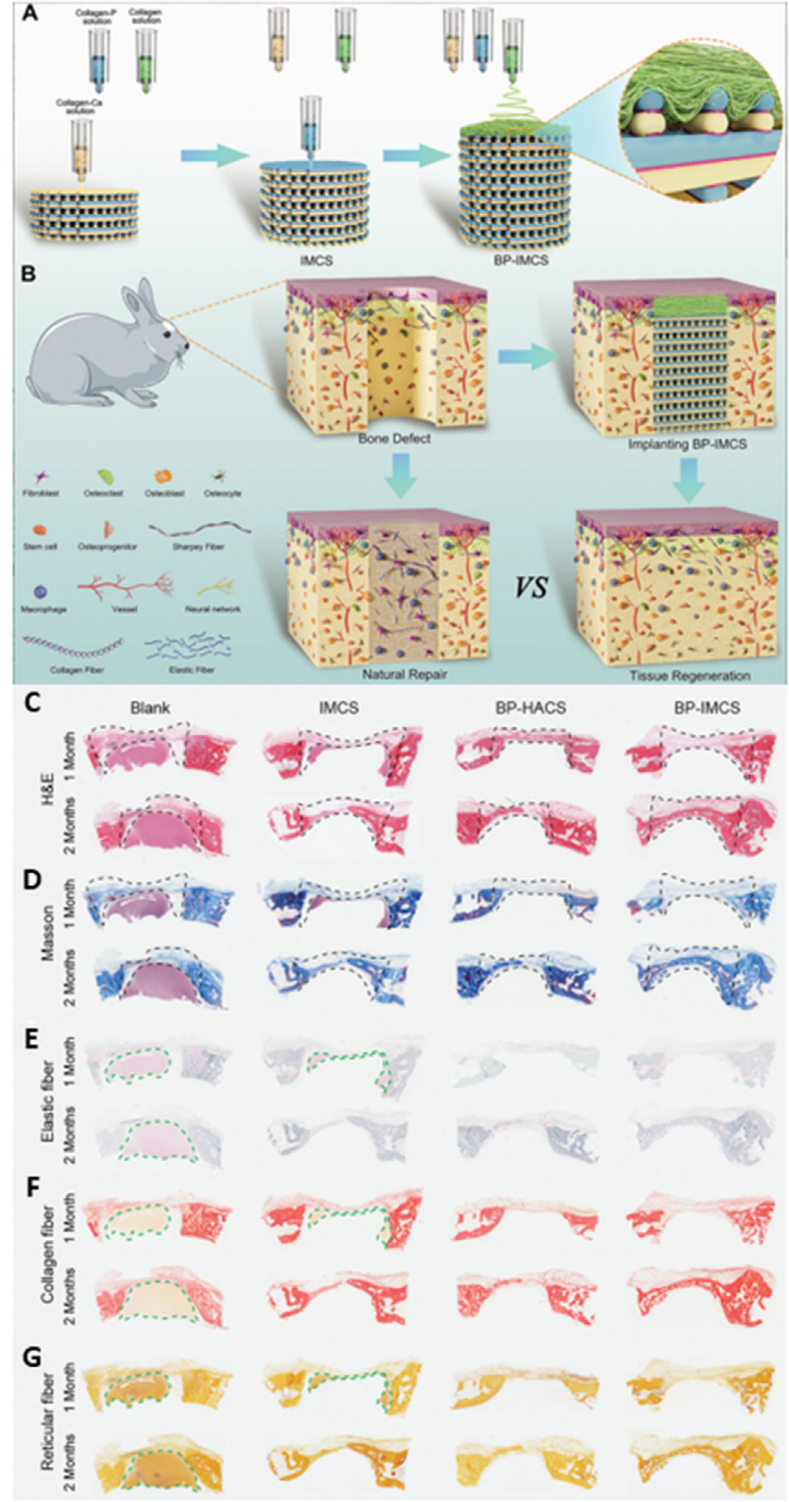
A) BP‐IMCS is developed through a continuous manufacturing strategy that combines 3D printing and electrospinning techniques. B) The efficacy of bone repair following the in vivo implantation of BP‐IMCS. Reproduced with permission. The staining of H&E A) and Masson B) was used to analyze the new bone formation, and the black box represented the area of bone regeneration. The staining of elastic fiber C), collagen fiber D), and reticular fiber E) was used to analyze preventive effect on fiber tissue invasion, and the green box represented the formed fibrous tissue.^[^ ^328^ ^]^ Copyright 2024, Wiley‐VCH.

The development of a bone membrane is a highly coordinated and orderly process, requiring the integration of structure and function to achieve natural membrane reconstruction and effective bone healing.^[^ ^24^, ^325^ ^]^ Wu et al. combined micro‐sol electrospinning technology with collagen nanofiber self‐assembly to fabricate a biomimetic bone membrane featuring a layered micro/nanofiber structure and sustained VEGF release. This design forms a physical bridge linking the cortical actin cytoskeleton with the extracellular matrix (ECM), allowing cells to sense various physical cues from the matrix. This microenvironment promotes mesenchymal cell survival, adhesion, proliferation, osteogenic differentiation, and the construction of endogenous formative layers. The biomimetic periosteum successfully repaired the bone defect and formed a dense, transparent connective tissue resembling the periosteum above the defect area. By eight weeks post‐surgery, early bone formation had developed into regular lamellar bone.^[^ ^326^ ^]^ Zhao et al. further advanced the field by designing a biomimetic bone membrane with an asymmetric structure using electrospinning. The randomly coaxial inner layer provides antioxidant properties that enhance cell recruitment, proliferation, differentiation, and mineralization, while the aligned outer layer facilitates angiogenesis and prevents fibroblast infiltration. This design precisely regulates inflammation, angiogenesis, and osteogenesis during bone repair.^[^ ^327^ ^]^ Despite these advancements, achieving gradient scaffolds with integrated and stable interfaces remains a challenge. To address this, Li et al. developed a bio‐inspired, collagen‐based biomimetic bone membrane scaffold through continuous 3D printing and electrospinning. This integrated scaffold (BP‐IMCS), composed of a biomimetic bone membrane (BP) and an in situ mineralized collagen scaffold (IMCS), demonstrated exceptional structural stability—tenfold greater than directly combined scaffolds. In vivo studies further confirmed that the BP‐IMCS significantly enhanced new bone formation by 32.47%, outperforming single‐layer scaffolds due to the synergistic effects of mineral ions and its biomimetic structure (**Figure** **9**A,B). BP facilitated bone regeneration by inhibiting the invasion of reticular fibers, thereby providing ample space for new bone growth. The morphology of the newly formed bone aligned with that of the rabbit skull, featuring an intermediate region of cancellous bone and a cortical bone surface (Figure 9C–G).^[^ ^328^ ^]^ Thus, biomimetic bone membranes with layered nanoscale structures hold great promise as advanced solutions for the treatment of bone defects.

The successful design and fabrication of artificial bone membranes hinge on the careful selection of scaffold materials, rational assembly strategies, and the accurate replication of the structure and function of natural bone membranes. Although significant progress has been made in developing various bone membrane materials, none have fully met the ideal requirements thus far. Further refinement of the macro‐ and microstructures of biomimetic bone membranes remains essential.

##### Lamellae and the Haversian System

Scaffolds for bone tissue engineering must emulate the natural layered extracellular matrix (ECM) and the intricate interfaces of bone tissue to effectively support regeneration. However, replicating the layered organization of bone ECM and its complex interfaces poses significant challenges. Dense bone is composed of tightly packed cylindrical subunits known as osteons or Haversian systems. Each osteon comprises 3 to 8 concentric lamellae encircling a central Haversian canal, which houses blood vessels and nerve fibers.^[^ ^329^, ^330^ ^]^ Developing biomimetic scaffolds that replicate the structural and physicochemical properties of bone ECM is therefore crucial.^[^ ^331^ ^]^

**Figure 10:**
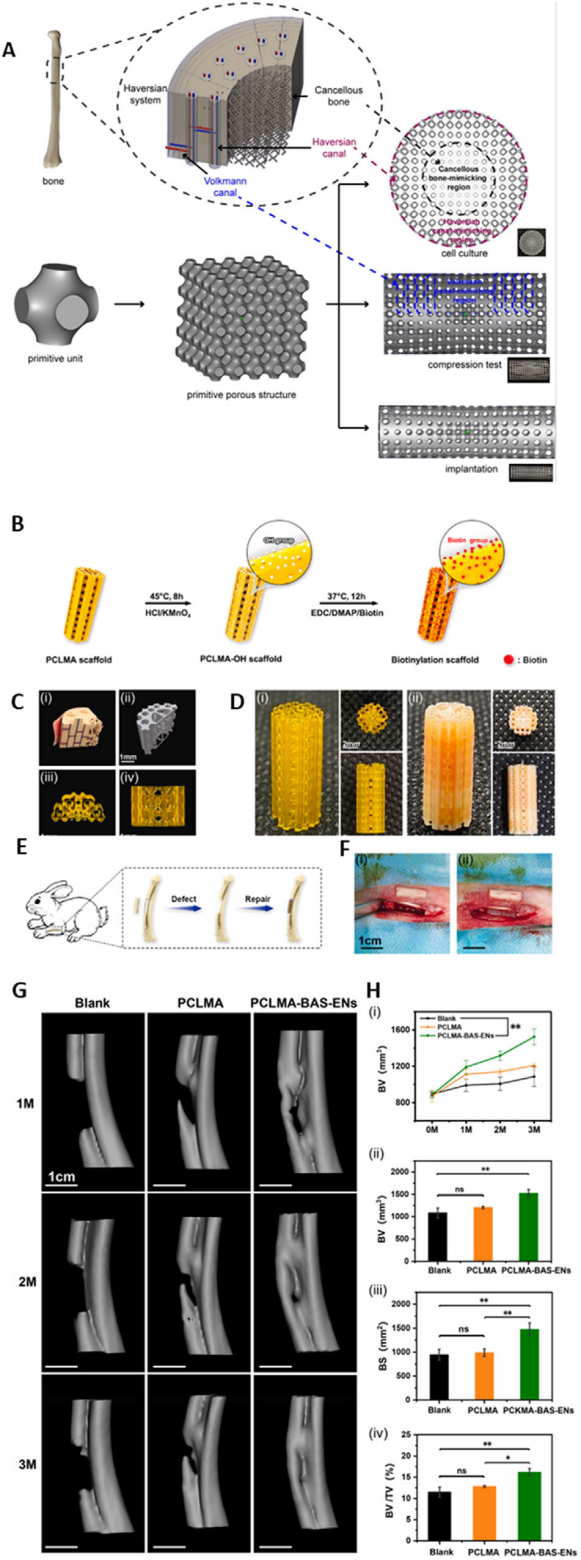
A) The diagrammatic sketch of using the primitive unit to construct different porous structures. Reproduced with permission.^[^ ^335^ ^]^ Copyright 2023, Elsevier. B) Schematic depiction of scaffold biotinylation. C) Pattern diagram of Haversian canal and the bionic scaffold (i‐ii), and the sectional view of imitated Haversian canal scaffold printed in PCLMA (iii‐iv). D) Biomimetic scaffold printed by DLP printer (i), and biotinylated scaffold (ii). Repair of radial bone defects in rabbits based on PCLMA‐BAS‐ENs scaffolds. E) Schematic diagram of animal surgery. F) Surgical procedures of implanting PCLMA‐BAS‐ENs scaffold (i‐ii). G) CT examination of regenerated bone tissue at 1 month, 2 months, and 3 months after implantation. H) Quantitative index of changes in the bone volume of experimental individuals (i). Quantitative indicators of bone volume (BV), bone volume/total volume (BV/TV), and bone surface area (BS) at three months (ii‐iv). (S–N–K test, *p < 0.05, and **p < 0.01). Data are expressed as mean ± SD (*n* = 5). Reproduced with permission.^[^ ^295^ ^]^ Copyright 2024, Elsevier.

Inspired by the unique architecture of bone, Sriram et al. designed a biomimetic scaffold consisting of an outer layer of 7% PHB/1% gelatin fibers (PG) mimicking cylindrical bone components such as osteons, with an inner layer of 7% PHB/0.5% polypyrrole (PPy) forming a double‐layer tubular structure. This scaffold simulates Haversian canals and supports anisotropic elongation of mouse BMSCs (mBMSCs), promoting osteogenic differentiation through the synergistic effects of physical and biochemical cues.^[^ ^332^ ^]^ However, tight nanofiber assembly during electrospinning can hinder cell migration. To address this, coral microparticles were adhered to the nanofiber layer, improving physical, mechanical, and cellular properties, and enhancing cell infiltration, proliferation, and mineralization.^[^ ^333^ ^]^ Building on biomimetic principles, Wei et al. developed a 3D scaffold with orthogonally oriented micro/nano‐layered structures featuring interconnected micropores and parallel nanosheets. The plate‐like hydroxyapatite nanosheets increased surface roughness and hydrophobicity, creating protein anchoring sites and promoting osteogenesis via activation of the Wnt/β‐catenin signaling pathway.^[^ ^334^ ^]^ Similarly, Li et al. employed the Triply Periodic Minimal Surface (TPMS) method to design a gradient porous scaffold resembling the Haversian system. This scaffold featured a gradual decrease in pore size, enabling the biomimetic recreation of the Haversian canal, Volkmann canals, and trabecular bone. The structure reduced stress shielding, improved mechanical strength, and facilitated bone ingrowth, osteoblast proliferation, and differentiation (**Figure** **10**A).^[^ ^335^ ^]^ To further advance scaffold design, Ghahri et al. developed a hollow, multilayer bioprinted scaffold using a four‐layer coaxial nozzle. This concentric structure mimicked the Haversian canal, with the inner layer containing endothelial cells and the outer layer hosting hMSCs. The scaffold exhibited suitable mechanical strength, stable swelling behavior, and optimal degradation rates, creating distinct niches for cell growth and differentiation.^[^ ^336^ ^]^ Jiang et al. have successfully manufactured high‐precision methylacrylylated polycaprolactone (PCLMA) bionic bone scaffold structures using 3D printing technology based on digital laser processing (DLP). Adipose‐derived stem cell‐engineered nanovesicles (ADSC‐ENs) were uniformly loaded onto the surface of the bionic scaffold using a perfusion device. The structural design of the scaffold, combined with the biological function of the nanovesicles, created a microenvironment conducive to tissue regeneration and long bone defect repair. In vivo CT imaging performed at the first, second, and third months revealed significant differences in bone defect repair status among the three groups. By the third month, most bone defects in the PCLMA‐BAS‐ENs group had been successfully repaired, with more complete and continuous bone integration on both sides of the defect area, accompanied by normal motor and activity function (Figure 10B–G).^[^ ^295^ ^]^

Cortical bone, as the functional unit of bone tissue, provides the blueprint for scaffold development. Biomimetic scaffolds that directly induce cortical bone formation could significantly accelerate postoperative recovery in orthopaedic surgery. While still in its infancy, research in this area holds promise for the development of novel therapies for orthopaedic reconstruction.

#### 5.1.2 Matching Structures

Advancements in X‐ray diffraction and electron microscopy have gradually unveiled the intricate structural details of both the organic and inorganic components of bone. The mineral phase of bone, primarily carbonate‐substituted hydroxyapatite, integrates into a pre‐formed, densely packed, and covalently cross‐linked organic matrix. Before mineralization occurs, the organic phase is already assembled, serving as a framework capable of precisely regulating crystal nucleation and growth.^[^ ^337^ ^]^ Notably, bone mineralization begins with nanoscale layering.^[^ ^20^ ^]^ This has spurred significant interest in the in vitro self‐assembly and mineralization of collagen, making it a focal point in bone tissue engineering (TE).

Early efforts to replicate the bone extracellular matrix (ECM) centered on the synchronized biomineralization process of collagen. This process begins with the precipitation of amorphous calcium phosphate (ACP) as collagen fibers self‐assemble.^[^ ^338^, ^339^, ^340^ ^]^ The ACP phase subsequently transforms into nanocrystalline hydroxyapatite, which deposits on the surface of collagen fibers.^[^ ^341^ ^]^ Shen et al. applied surface‐oriented epitaxial crystallization on 3D‐printed polycaprolactone (PCL) scaffolds, creating an adaptive nanoscale morphology in the form of PCL sheets that provided precise control over hydroxyapatite nucleation. This approach generated a microenvironment resembling the natural ECM, demonstrating excellent osteogenic potential and significantly accelerating in vivo bone regeneration.^[^ ^105^ ^]^ Despite these advancements, replicating the complex biomechanical and biochemical properties of natural ECM remains a significant challenge in fabricating artificial matrices.^[^ ^342^ ^]^ Xie et al.^[^ ^343^ ^]^ addressed this by incorporating hard peptide fibers into a hydrogel network using dynamic imine bonds. This resulted in fibrous hydrogels with advantageous properties such as heterogeneous structures, macro stability, cell‐adaptive network dynamics, and sustained delivery of biochemical molecules, effectively mimicking the biophysical and biochemical characteristics of natural ECM. Additionally, the dynamic adaptive network facilitated intercellular interactions, enhancing cellular metabolism through the E‐cadherin/AMPK mechanism.^[^ ^344^, ^345^, ^346^ ^]^ Notably, activated AMPK plays a critical role in enhancing cellular mechanotransduction and promoting YAP/TAZ nuclear localization, further supporting osteogenesis.^[^ ^344^, ^347^ ^]^

**Figure 11:**
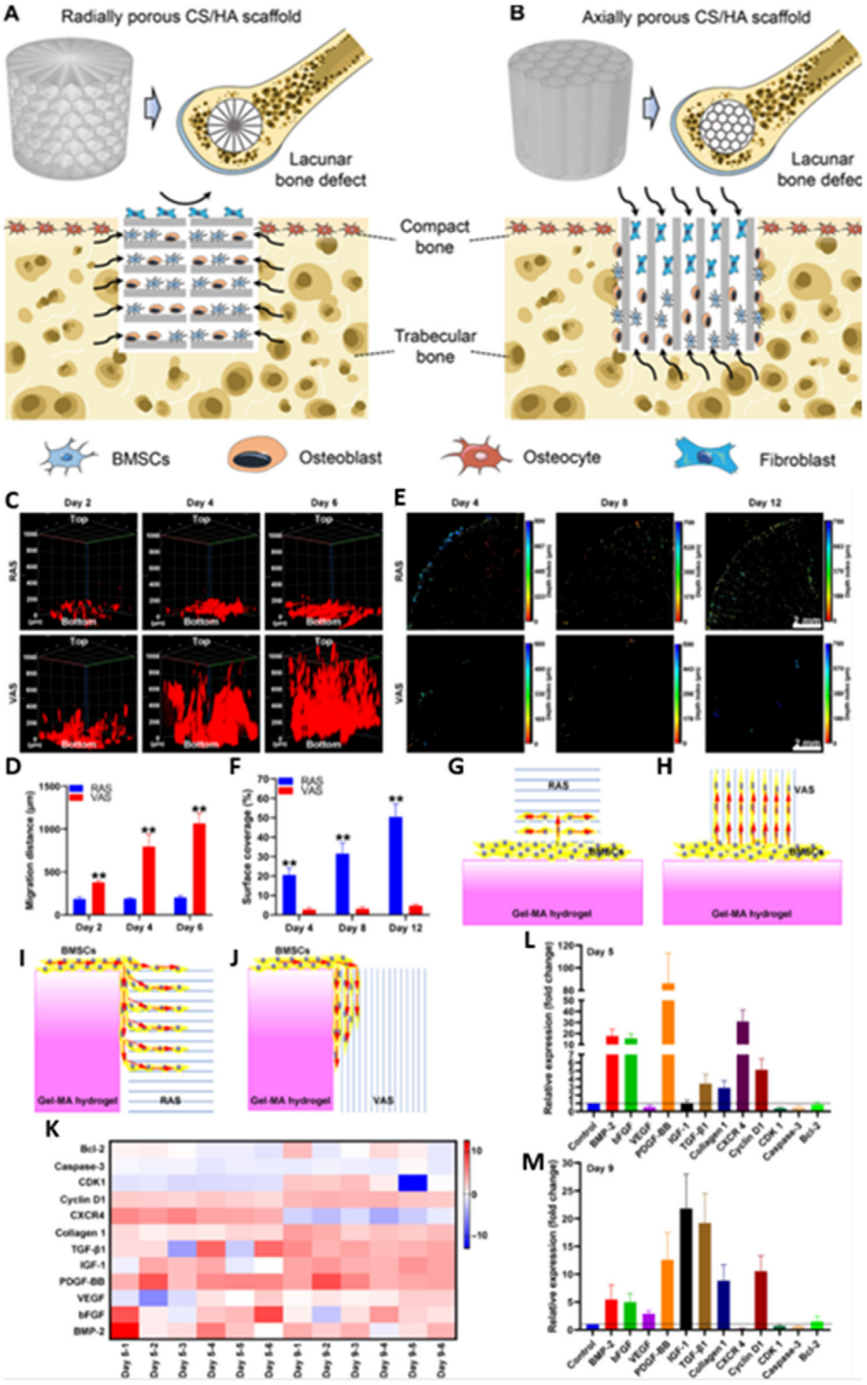
A) Radially porous scaffolds can guide resident bone repair‐related cells from their peripheral pore channels (indicated by curved arrows) to the interior while preventing unrelated cell invasion into the scaffold. B) Axially porous scaffolds allow resident bone repair‐related cells to migrate from their bottom pore channels (indicated by curved arrows) while also allowing fibroblasts to infiltrate. C) Fluorescence images show BMSCs migrating from the bottom to the top of RAS and VAS scaffolds. D) Quantification of BMSCs migration distance from the bottom to the top of RAS and VAS. E) Fluorescence images display the distribution of migrating BMSCs from the surrounding area to the centers of RAS and VAS. F) The surface coverage of BMSCs migrating towards RAS and VAS. G,H) Schematic illustrations of potential migration routes of BMSCs from the bottom to the top of RAS and VAS. I,J) Schematic illustrations of potential migration routes of BMSCs from the surrounding area to the centers of RAS and VAS. K) Heat map displaying the relative expression of BMP‐2, bFGF, VEGF, PDGF‐BB, insulin‐like growth factor 1 (IGF‐1), transforming growth factor‐β (TGF‐β1), collagen I, CXCR4, cyclin D1, CDK1, caspase‐3, and BCL‐2 in BMSCs cultured in RAS for 5 and 9 days. Blue represents downregulation, and red represents upregulation. L) and M) Relative expression of BMP‐2, bFGF, VEGF, PDGF‐BB, IGF‐1, TGF‐β1, collagen I, CXCR4, cyclin D1, CDK1, caspase‐3, and BCL‐2 in BMSCs cultured in RAS for 5 and 9 days. **P < 0.01. Reproduced with permission.^[^ ^351^ ^]^ Copyright 2022, Wiley‐VCH.

Mineralized collagen fibers (MCFs), as nanoscale building blocks of bone, are critical to the mechanical properties of bone due to the anisotropic and ordered interactions between collagen and hydroxyapatite (HAp).^[^ ^319^ ^]^ These anisotropic and ordered structures promote osteogenesis through multiple mechanisms: i) Enhanced diffusion and permeability: Transforming traditional electrospun nanofiber mats into 3D constructs with hierarchical structures and controlled alignment, such as RAS and VAS (**Figure** **11**), facilitates bone marrow stem cell (BMSC) migration.^[^ ^348^, ^349^, ^350^, ^351^ ^]^ These structures guide BMSC migration along the fiber alignment, from the periphery to the center and from bottom to top. This directed migration is likely linked to the downregulation of Zyxin protein expression in BMSCs,^[^ ^352^ ^]^ which reduces adhesion spot size and dynamic traction forces, enabling faster and more directed cell movement. ii) Regulation of cell morphology, cytoskeletal tension, and orientation: Cytoskeletal reorganization and nuclear elongation are vital for signal transduction during cell differentiation. Studies have associated cytoskeletal and nuclear elongation with altered gene expression patterns and cell fate decisions.^[^ ^353^, ^354^ ^]^ Research shows that human mesenchymal stem cells (hMSCs) align with the morphology of underlying nanofibers. While hMSCs on randomly arranged nanofibers adopt a polygonal shape and are randomly distributed, hMSCs on aligned nanofibers exhibit a spindle‐shaped morphology, stretching along the fiber's long axis.^[^ ^355^ ^]^ These findings underscore the importance of nanofiber alignment in influencing cell behavior and osteogenesis.

Despite numerous studies on collagen self‐assembly and mineralization induction, effectively controlling the self‐assembly process and calcium secretion patterns remains challenging. Issues such as suboptimal fiber architecture and the random formation and distribution of calcium significantly hinder the development of more precise and efficient strategies for bone repair.

### 5.2 Constructing the Bionic Vascular System

An adequate blood supply, essential for transporting oxygen and nutrients while eliminating metabolic waste, is critical for effective bone fracture repair. Most mesenchymal stem cells (MSCs) in bone tissue reside around H‐type blood vessels, whose endothelial cells (ECs) play a pivotal role in coordinating MSC self‐renewal and differentiation. This coordination occurs through the secretion of vascular factors such as FGFs, PDGFs, and BMPs, as well as paracrine signals like Notch signaling. These processes not only regulate bone development and maintain homeostasis but also contribute significantly to the regeneration of bone defects.^[^ ^356^, ^357^, ^358^, ^359^, ^360^ ^]^ The following discussion focuses on the delivery of angiogenic factors and strategies for promoting vascularization.

#### 5.2.1 Pre‐Vascularization Approaches for Bone Regeneration

In large‐volume bone defects, especially those exceeding the critical size, persistent ischemia and hypoxia at the defect's center pose significant challenges, often resulting in nonunion.^[^ ^361^, ^362^ ^]^ Beyond loading scaffolds with angiogenic factors and controlling their release profiles, scaffolds can also be designed to intrinsically promote vascular formation. In vitro endothelial cell vascularization has emerged as a promising approach to enhance the vascularization of implanted scaffolds in vivo.^[^ ^363^, ^364^ ^]^ Incorporating hollow channels and pre‐vascularization into scaffold design is an effective strategy to improve vascular formation and perfusion in large grafts. For instance, Park et al. utilized 3D printing technology to fabricate a pre‐vascularized scaffold capable of temporally and spatially enhancing the repair of large‐volume bone defects. Hypoxic conditions at the scaffold's center triggered rapid neovascularization mediated by VEGF.^[^ ^365^ ^]^

Nanomaterial‐based cell sheet (CS) technology represents an innovative approach with significant potential.^[^ ^366^ ^]^ This method can reconstruct a vascularized bone microenvironment without relying on scaffold support. Notably, MSC sheets ≈300 µm thick, composed of 10–15 layers of cells, have been successfully fabricated using magnetic nanoparticles (MNPs).^[^ ^366^, ^367^ ^]^ Studies in mice demonstrated that MSC sheets enhanced angiogenesis primarily through the release of angiogenic factors rather than direct endothelial cell differentiation.^[^ ^368^ ^]^ Additionally, Silva et al. developed a layered 3D vascularized network that promotes cell adhesion, proliferation, and interconnectivity, supporting the deposition of mineralized matrices and the formation of layered bone micro‐tissues under in vitro conditions.^[^ ^369^ ^]^ This technology holds promise for creating robust, multicellular, and reproducible structures for bone tissue engineering. However, its limitations in replicating the complexity of the bone microenvironment must be addressed. Furthermore, integrating biophysical cues provided by biomaterials is essential for forming a functional microvascular system in bone tissue.

Furthermore, alternative vascularization strategies have been proposed to enhance bone regeneration. Bjorge et al.^[^ ^48^ ^]^ introduced a modular and layered tissue engineering approach to create bone‐like tissues embedded with signals that promote vascularization. This innovative strategy integrated multiple factors, including groove morphology, cell culture conditions, static versus dynamic environments, and the impact of biochemical stimuli, demonstrating a high degree of control at the initial stages. While lumen formation was not observed, interactions between cells and the deposited ECM suggested the potential for pre‐vascular structure development. Notably, nanogrooved morphology significantly increased the presence of key proteins and signals in cell microvesicle secretion, such as Wnt signaling, which plays a critical role in endothelial differentiation and the stabilization of immature vascular systems.^[^ ^370^ ^]^

Reconstructing a stable and functional vascular network in vitro while preserving its integrity and functionality post‐implantation remains a significant challenge. Future advancements are expected to emphasize the integration of the physical, mechanical, and biochemical properties of biomaterials to create an optimal microenvironment for vascularization, benefiting both transplanted and resident bone cells. Achieving this goal will require targeted improvements in key parameters that promote microvascular formation, stability, and functionalization.

#### 5.2.2 Delivery of Angiogenic Factors

During natural bone healing, angiogenesis plays a critical role throughout most of the process, except during the initial inflammatory phase. This complex process is regulated by multiple angiogenic factors, which must be delivered in alignment with their natural release patterns for optimal effect. Typically, the mode of delivery is dictated by the scaffold's degradation characteristics, which can be tailored through adjustments to scaffold composition, morphology, and drug encapsulation techniques.^[^ ^371^, ^372^, ^373^ ^]^ Importantly, each growth factor can perform diverse functions within the regenerative niche. The intricate crosstalk between growth factors and key cell types further underscores the limitations of current scaffolds in replicating the full complexity of the natural healing process.^[^ ^372^ ^]^

**Figure 12:**
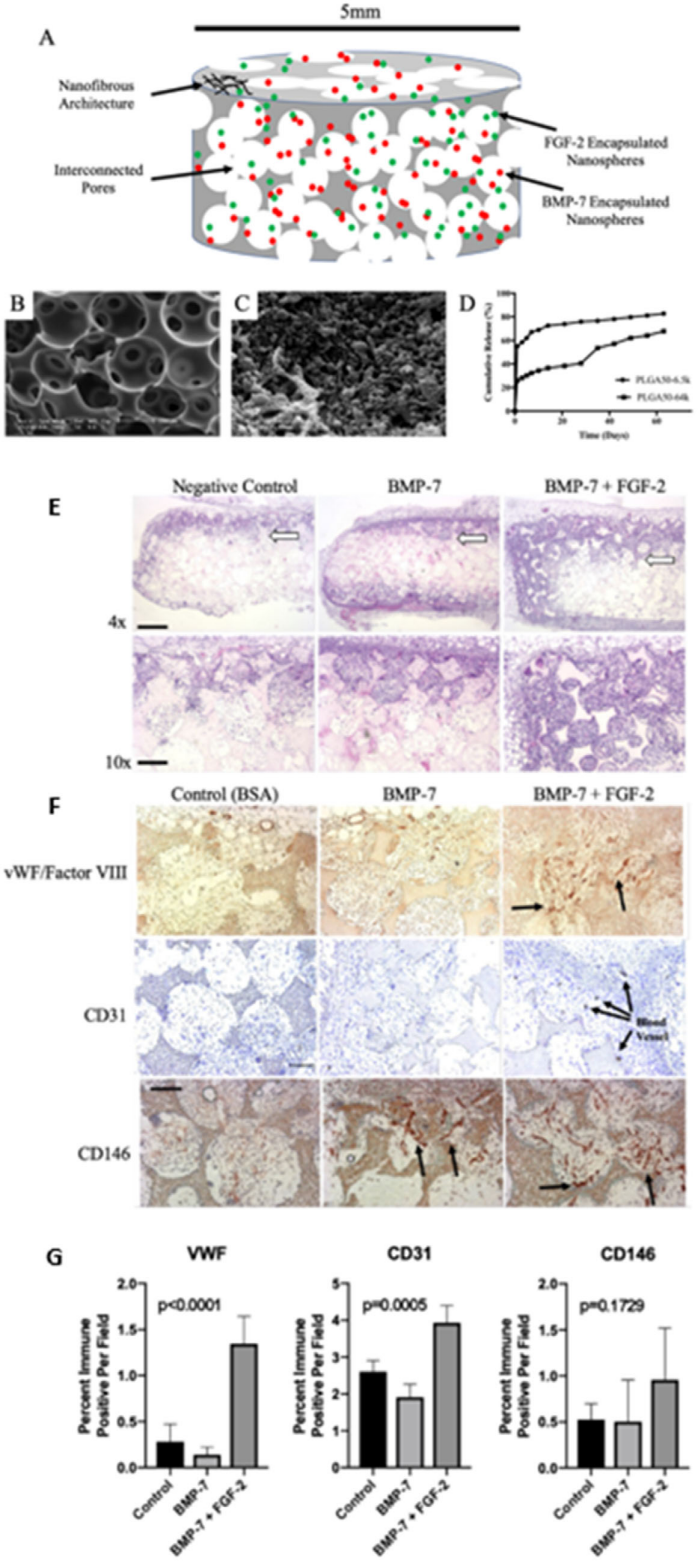
Dual‐release of BMP‐7 and FGF‐2 on 3D PLLA nanofibrous scaffolds. A) Schematic of FGF‐2‐PLGA nanospheres and BMP‐7‐PLGA nanospheres immobilized on PLLA nanofibrous scaffolds. B) The morphology of the PLLA nanofibrous scaffold after the incorporation of PLGA nanospheres was observed with SEM (low magnification). C) High magnification of SEM to observe the morphology of PLGA nanospheres immobilized PLLA nanofibrous scaffold. D) Cumulative release curve for FGF‐2 released from low or high molecular weight PLGA nanospheres incorporated into PLLA scaffolds in vitro. E) FGF‐2‐containing nanospheres in nanofibrous scaffolds stimulate tissue invasion in vivo, where white arrows indicate the boundaries between tissue‐invaded and uninvaded areas in the implanted scaffolds. The upper panel is at low magnification (4X) (scale bar = 500 µm). The lower panel is magnified higher (10X) (scale bar = 200 µm). F) Early vascularization was visualized using Factor VIII‐related antigen/von Willebrand Factor (upper panel), CD31 (middle panel), and CD146 (lower panel) immunohistochemical staining (scale bar = 50 µm, the same magnification for all three panels). G) The immunohistochemical stain was quantified using ImageJ. Reproduced with permission.^[^ ^375^ ^]^ Copyright 2024, Elsevier.

Simultaneously controlling the release of two growth factors, known as “dual release,” offers a promising approach to closely replicate the natural healing process. Fang et al. developed a scaffold by integrating electrospun polycaprolactone (PCL) fibers with electro‐sprayed poly(lactic‐co‐glycolic acid) (PLGA) microspheres encapsulating Spp1 and Cxcl12. This design enabled precise local delivery of both factors, promoting angiogenesis through the coordinated activation of the MAP kinase pathway, thereby enhancing vascular restoration and accelerating bone regeneration.^[^ ^374^ ^]^ Building on this concept, Rambhia et al. created PLGA nanospheres for the independent and controlled release of bone morphogenetic protein‐7 (BMP‐7) and basic fibroblast growth factor (FGF‐2), which were incorporated into a poly(L‐lactic acid) (PLLA) nanofiber scaffold. The release of FGF‐2 enhanced stem cell migration, proliferation, and angiogenesis, optimizing BMP‐7‐induced bone regeneration through carefully tuned dosage and kinetics (**Figure** **12**).^[^ ^375^ ^]^ While traditional chemically cross‐linked hydrogels often exhibit static properties, cells in vivo interact with dynamic microenvironments that influence their behavior, physicochemical properties, and fate.^[^ ^376^, ^377^ ^]^ To address this, Miao et al. designed a bioactive gel scaffold by integrating black phosphorus nanosheet‐supported dynamic deoxyribonucleic acid (DNA) hydrogels with a 3D‐printed scaffold. The 2D black phosphorus nanosheets (BPNSs) formed nanoscale physical networks with large DNA chains through non‐covalent interactions, reinforcing the hydrogel mechanically while enabling vascular endothelial growth factor (VEGF) loading. This combination enhanced osteogenesis and supported the formation of mature blood vessels, achieving significant bone regeneration.^[^ ^378^ ^]^ Furthermore, Li et al. developed a composite dynamic hydrogel composed of gelatin methacrylate (GelMA) and a DNA interpenetrating polymer network, functionalized with Apt19S and VEGF aptamers (AptV). This hydrogel promoted BMSC adhesion and provided a sustained VEGF release platform, activating the focal adhesion kinase (FAK)/phosphatidylinositol 3‐kinase (PI3K)/protein kinase B (Akt)/β‐catenin signaling pathway. These mechanisms collectively enhanced vascularized bone regeneration.^[^ ^379^ ^]^

However, therapies involving angiogenic factors have yielded inconsistent results, particularly regarding their efficacy in promoting in vivo vascularization and osteogenesis.^[^ ^380^, ^381^ ^]^ These discrepancies are largely attributed to the limited understanding of the underlying mechanisms in critical‐sized bone defects. Future research should focus on identifying and elucidating the roles of key biomolecules involved in the intricate interplay between angiogenesis and osteogenesis. A deeper understanding of these mechanisms could pave the way for more effective applications in bone tissue engineering.

### 5.3 Constructing the Bone Immune Microenvironment

The bone immune microenvironment plays a multifaceted role within the stem cell niche, encompassing the recruitment of stem cells and their subsequent osteogenic differentiation. Mesenchymal stem cells (MSCs) are highly responsive to the local inflammatory microenvironment, producing a diverse array of cytokines and growth factors that facilitate tissue repair. The immunoregulatory plasticity of MSCs has thus become a focal point in bone regenerative medicine.^[^ ^382^ ^]^ A new generation of immunomodulatory scaffolds, currently under development for bone tissue regeneration, is designed through precise control of scaffold morphology, surface topography, chemical composition, and incorporation of bioactive proteins.^[^ ^383^, ^384^, ^385^, ^386^ ^]^

**Figure 13:**
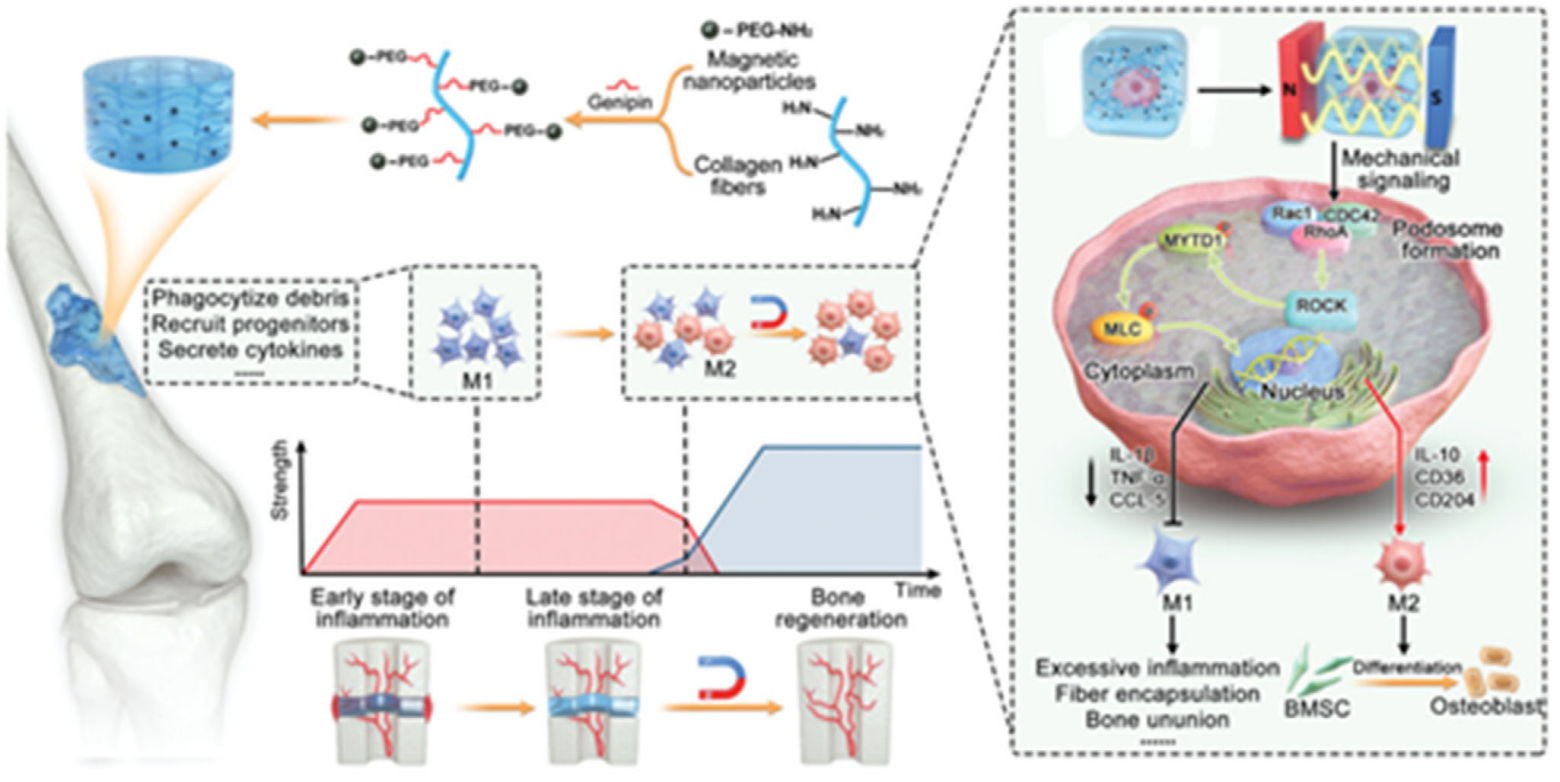
Schematic illustration of magnetized nanocomposite hydrogels for on‐demand immunomodulation via temporally controlled macrophage phenotypic transition in response to a magnetic field. Fabrication processes for the magnetized hydrogels and the scheduled inflammation regulation strategy through timely programmed macrophage phenotypic switching from M1 to M2 polarization under the manipulation of a magnetic field. M1/ M2, M1/M2 polarized macrophage; BMSC, bone mesenchymal stem cell. Reproduced with permission.^[^ ^388^ ^]^ Copyright 2022, Wiley‐VCH.

It is well established that immune cells, including macrophages, neutrophils, and B cells, contribute significantly to bone tissue repair, with macrophages playing a particularly prominent role. Macrophages polarize into either pro‐inflammatory M1 or anti‐inflammatory M2 phenotypes, depending on environmental stimuli.^[^ ^387^ ^]^ Enhancing the differentiation of macrophages into the M2 phenotype has emerged as a promising strategy to create an immune microenvironment conducive to osteogenesis. For instance, Zhang et al. developed peptides with M2 regulatory and self‐assembly modules as building blocks for constructing ultrasound‐responsive nanofiber hydrogels. These nanofibers release gradually under ultrasound stimulation, activating mitochondrial glycolytic metabolism and the tricarboxylic acid cycle, thereby suppressing reactive oxygen species production and enhancing M2 macrophage polarization. The hydrogel also accelerates the differentiation of bone marrow mesenchymal stem cells (BMSCs) into osteoblasts by inducing M2 macrophages to secrete BMP‐2 and IGF‐I.^[^ ^172^ ^]^ Similarly, Huang et al. grafted superparamagnetic α‐Fe₂O₃/γ‐Fe₂O₃ nanoparticles onto collagen nanofibers, creating a platform for mechanical stimulation. This system applies micro/nano forces to macrophages under a magnetic field, activating the podosome/Rho/ROCK mechanical signaling pathway and promoting the transition from M1 to M2 phenotypes (**Figure** **13**).^[^ ^388^ ^]^ In addition to macrophage modulation, monocytes play a critical role in angiogenesis and osteogenesis within bone tissue engineering. Sun et al. demonstrated that silicified collagen nanofibers enhance monocyte recruitment, differentiation, and cytokine release, further attracting MSCs and endothelial progenitor cells. This approach promotes local vascularization and bone regeneration.^[^ ^389^, ^390^ ^]^

The surface nanoscale morphology of biomaterials also influences the bone immune microenvironment by modulating local immune responses. Joanna et al. reported that nanostructured needle‐like biomimetic calcium‐deficient hydroxyapatite (CDHA) facilitates osteoblast activity while inducing macrophage release of pro‐inflammatory cytokines. The porosity of CDHA substrates modulates the intensity of the inflammatory response, underscoring the sensitivity of macrophages to the structural characteristics of biomaterials.^[^ ^391^ ^]^ Similarly, Jin et al. demonstrated that biomimetic layered nanosurfaces stimulate macrophages, promoting M2 polarization and interleukin‐4 secretion, which subsequently enhance stem cell osteogenesis and endogenous bone regeneration.^[^ ^106^ ^]^ In addition, a recent study has demonstrated that mesoporous silica nanoparticles (MSN) with a pollen‐like surface morphology (PMSN) exhibit enhanced physical contact with macrophage surfaces compared to MSN with smooth surface morphology. This interaction activates the ERK signaling pathway by upregulating CD28, thereby promoting the phenotype transition of M1 macrophages to M2 macrophages, which in turn facilitates bone regeneration.^[^ ^392^ ^]^

In summary, a deeper understanding of the potential synergistic regulation within this environment is crucial for designing new immunomodulatory strategies that can create an ideal regenerative niche for bone regeneration. This interplay among immune cells, bone‐affiliated cells, and stem cells is pivotal in bone repair processes. Future studies should focus on developing advanced stimulus‐responsive materials to investigate the immune regulatory functions of macrophages, with the aim of elucidating the specific effects of biophysical stimuli.

### 5.4 Combination Between Systems

Different niches interact and are intricately interconnected. Integrating multiple niches is a critical aspect of designing comprehensive bone tissue scaffolds that replicate the composition and architecture of natural bone tissue, thereby creating a microenvironment that closely resembles in vivo conditions.

Zhang et al. developed a biomimetic layered scaffold comprising deferoxamine@poly(ε‐caprolactone) nanoparticles (DFO@PCL NPs), carbonyl manganese (MnCO) nanosheets, gelatin methacrylate hydrogels, and poly(lactic‐co‐glycolic acid)/hydroxyapatite (HA) matrices. This 3D‐printed scaffold features a well‐organized gradient structure, mimicking both cortical and trabecular bone tissues, with soft hydrogel infusion providing extracellular matrix‐like characteristics. At the implantation site, a Fenton‐like reaction between MnCO and endogenous hydrogen peroxide enables the sustained release of carbon monoxide and Mn^2^⁺, significantly mitigating inflammation by promoting M2 macrophage polarization. These macrophages secrete vascular endothelial growth factor (VEGF), inducing vascular formation. Results demonstrated that VEGF secretion synergized with DFO@PCL NPs to promote angiogenesis via the Mn^2^⁺‐mediated activation of the HIF‐1α pathway. The scaffold exhibited strong immunomodulatory properties, reducing inflammation, enhancing vascularization, limiting osteoclast formation, and significantly improving osteogenesis, offering considerable potential for advancing bone regeneration.^[^ ^393^ ^]^ Additionally, Cui et al. employed a dual‐model bioprinting technique to fabricate composite scaffolds. A high‐temperature nozzle was used to print a porous polycaprolactone composite with nanotricalcium phosphate (PCL‐nTCP) as the supporting framework, while a temperature‐controlled light‐curing nozzle printed a hydrogel loaded with endothelial cells to create biomimetic vasculature. The hydrogel, enriched with concentrated growth factors (CGF), facilitated the slow release of biological signals, promoting angiogenesis and osteogenic differentiation in bone marrow mesenchymal stem cells (BMSCs). The scaffold exhibited excellent mechanical properties and porosity, supporting tissue regeneration.^[^ ^394^ ^]^ These studies highlight innovative strategies for biomimetic scaffold design, paving the way for efficient bone tissue regeneration.

The development of bioengineered scaffolds with intricate complexity and diverse niches represents a crucial avenue for future bone regeneration research. However, achieving the precise design and integration of these distinct niches remains a significant challenge.

## 6 Conclusion and Outlook

Designing stem cell niches with high biological complexity and functionality for bone regeneration—encompassing processes like osteogenesis, angiogenesis, and immunomodulation—remains a significant challenge in bone tissue engineering. Advancing our understanding of stem cell niches, cell‐scaffold interactions, and the heterogeneity of mesenchymal stem cells (MSCs) is crucial. While osteoblasts are traditionally thought to arise from MSCs, these cells exhibit notable heterogeneity, encompassing various subpopulations within uncharacterized cultures.^[^ ^395^ ^]^ To date, comprehensive grading and separation of MSC subpopulations with definitive stem cell traits, such as self‐renewal and in vivo pluripotency, have yet to be systematically conducted. Future efforts must focus on isolating and characterizing MSC subtypes to enable the creation of personalized stem cell niches. Furthermore, integrating cutting‐edge stem cell technologies, including genetic modification, could mitigate premature cell death and further enhance bone regeneration outcomes.

Safety remains a critical concern in the application of nanomaterials within the human body. Despite their immense potential in bone tissue engineering, the precise mechanisms underlying the immunological and toxicological effects of nanomaterials remain inadequately understood. It is therefore essential to investigate how nanomaterial properties—such as size, shape, and surface chemistry—interact with biological systems, as nanoscale structures can elicit unintended adverse reactions in surrounding tissues.^[^ ^396^ ^]^ Promisingly, emerging computational tools in biotechnological research, including artificial intelligence (AI) and deep machine learning,^[^ ^397^ ^]^ offer the capability to integrate the complex attributes of nanomaterials with data on immune responses and immunotherapies. These advancements hold the potential to significantly accelerate the development of safer and more effective bone tissue engineering strategies.

The integration of physical, mechanical, and biochemical signals within stem cell niches represents a promising direction for the future of bone tissue engineering. There is still a clinical need for bionic dense bone materials that possess both high strength and toughness. While these materials offer structural support, they lack adequate space for osteoblast migration and internal regeneration. Moreover, achieving an optimal balance between material degradation rates and new bone formation poses a significant challenge. Through the recruitment of stem cells, promotion of their proliferation and differentiation, and subsequent secretion of mineralized bone matrix, the simulation of a stem cell niche facilitates the generation of bone tissue that closely resembles natural bone. This approach not only achieves excellent mechanical strength but also offers enhanced long‐term stability and functional maintenance of regenerated bone compared to conventional bionic methods. Attaining an optimal balance between structures that support stem cell growth and those that fully replicate mature bone characteristics—such as mechanical strength—requires additional investigation. Currently, developing perfect biomimetic nanomaterials capable of meeting such requirements remains challenging. Therefore, interdisciplinary efforts spanning chemistry, materials science, mechanics, physics, biology, and other disciplines are essential for developing more intricate and adaptable nanomaterials featuring diverse nanostructures. Beyond the mechanical and biochemical signals discussed in this article, recent studies highlight the potential of physiological cues—such as electrical, magnetic, acoustic, thermal, and optical stimuli—in influencing stem cell behavior and promoting cellular maturation events critical to skeletal regeneration.^[^ ^398^ ^]^ In conclusion, substantial progress has been made in recent years toward enhancing bone regeneration strategies through biomimetic materials that emulate stem cell niches. The development of such advanced biomimetic nanoplatforms offers a compelling and transformative approach to the future of bone tissue engineering.

## Conflict of Interest

The authors declare no conflict of interest
